# The thyroid hormone enhances mouse embryonic fibroblasts reprogramming to pluripotent stem cells: role of the nuclear receptor corepressor 1

**DOI:** 10.3389/fendo.2023.1235614

**Published:** 2023-12-01

**Authors:** Constanza Contreras-Jurado, Ana Montero-Pedrazuela, Raúl F. Pérez, Susana Alemany, Mario F. Fraga, Ana Aranda

**Affiliations:** ^1^ Instituto de Investigaciones Biomédicas Sols-Morreale (IIBM), Consejo Superior de Investigaciones Científicas (CSIC)-Universidad Autónoma de Madrid (UAM), Madrid, Spain; ^2^ Departamento de Bioquímica, Facultad de Medicina, Universidad Alfonso X El Sabio, Madrid, Spain; ^3^ Centro de Investigación Biomédica en Red de Cáncer (CIBERONC), Instituto de Salud Carlos III, Madrid, Spain; ^4^ Cancer Epigenetics and Nanomedicine Laboratory, Centro de Investigación en Nanomateriales y Nanotecnología (CINN), CSIC-UNIOVI-Principado de Asturias, Oviedo, Spain; ^5^ Health Research Institute of Asturias (ISPA), Oviedo, Spain; ^6^ Institute of Oncology of Asturias (IUOPA), University of Oviedo, Oviedo, Spain; ^7^ Department of Organisms and Systems Biology (BOS), University of Oviedo, Oviedo, Spain; ^8^ CIBER of Rare Diseases (CIBERER), Oviedo, Spain

**Keywords:** thyroid hormone, mouse embryonic fibroblasts, reprogramming, pluripotency, nuclear receptor corepressor 1

## Abstract

**Introduction:**

Pluripotent stem cells can be generated from somatic cells by the Yamanaka factors Oct4, Sox2, Klf4 and c-Myc.

**Methods:**

Mouse embryonic fibroblasts (MEFs) were transduced with the Yamanaka factors and generation of induced pluripotent stem cells (iPSCs) was assessed by formation of alkaline phosphatase positive colonies, pluripotency gene expression and embryod bodies formation.

**Results:**

The thyroid hormone triiodothyronine (T3) enhances MEFs reprogramming. T3-induced iPSCs resemble embryonic stem cells in terms of the expression profile and DNA methylation pattern of pluripotency genes, and of their potential for embryod body formation and differentiation into the three major germ layers. T3 induces reprogramming even though it increases expression of the cyclin kinase inhibitors *p21* and *p27*, which are known to oppose acquisition of pluripotency. The actions of T3 on reprogramming are mainly mediated by the thyroid hormone receptor beta and T3 can enhance iPSC generation in the absence of c-Myc. The hormone cannot replace Oct4 on reprogramming, but in the presence of T3 is possible to obtain iPSCs, although with low efficiency, without exogenous Klf4. Furthermore, depletion of the corepressor NCoR (or Nuclear Receptor Corepressor 1) reduces MEFs reprogramming in the absence of the hormone and strongly decreases iPSC generation by T3 and also by 9cis-retinoic acid, a well-known inducer of reprogramming. NCoR depletion also markedly antagonizes induction of pluripotency gene expression by both ligands.

**Conclusions:**

Inclusion of T3 on reprogramming strategies has a potential use in enhancing the generation of functional iPSCs for studies of cell plasticity, disease and regenerative medicine.

## 1 Introduction

Somatic cells can be reprogrammed to induced pluripotent stem cells (iPSCs) with the introduction of the Yamanaka factors (Oct4, Sox2, Klf4 and c-Myc or OSKM) (1, 2), which can also induce totipotency *in vivo* (3). Herein, reprogramming refers to the reversion of somatic cells into an embryonic stem (ES) cell-like state. As iPSCs have the ability of self-renewal and the potential to differentiate into virtually any type of somatic cell, they provide an invaluable tool for the study of cell plasticity and have revolutionized the fields of regenerative medicine, tissue engineering and drug screening (4–6). iPSCs are genetically similar to ES cells and express ES cell-specific markers, although they can present genetic and epigenetic abnormalities (7, 8). Reprogramming is dominated by a small number of “elite” clones that are especially poised to become pluripotent stem cells (9, 10). To be converted into iPSCs, cells must first exit the differentiated state undergoing a mesenchymal to epithelial transition associated to down-regulation of somatic genes (11, 12). Cells then progress through an intermediate state in which they activate embryonic markers such as alkaline phosphatase (AP), and finally express endogenous core pluripotency genes including *Oct4*, *Sox2* and *Nanog*, acquiring a stable self-sustaining pluripotent immortal state, which no longer requires exogenous Yamanaka factors (13–15). This process requires a largescale epigenetic modelling. Besides DNA demethylation of pluripotency genes, which is required for reprogramming, pluripotent cells exhibit global DNA hypomethylation, a pattern that may indicate an open chromatin state (16–19). Accordingly, iPSCs, compared with somatic cells, are enriched for histone modifications associated with active chromatin such as histone acetylation, and depleted for histone methylation marks characteristic of transcriptional repression, suggesting that pluripotent cells are more euchromatic than somatic cells (20). Thus, chromatin structure is a key controller of reprogramming and several studies have demonstrated the importance of different epigenetic regulators in converting somatic cells into iPSCs (21, 22).

The thyroid hormones thyroxine (T4) and triiodothyronine (T3) are crucial regulators of growth, development and metabolism (23). The actions of the active thyroid hormone T3 are initiated by binding to the thyroid hormone receptors (TRs), which belong to the superfamily of nuclear receptors (24). Two different genes encode TRα (*Nr1a1 or THRA*) and TRβ (*Nr1a2 or THRB*) proteins, respectively, which show different expression pattern and functions (25). TRs, as well as other nuclear receptors, bind their response elements on target genes preferentially as heterodimers with another nuclear receptor, the retinoid X receptor (RXR). RXR binds the vitamin A derivative 9-cis-retinoic acid (9c-RA) and other selective ligands known as rexinoids (26), which can play an active role in signaling by some of their partner receptors (24). 9c-RA can also bind the retinoic acid receptors (RARs), which are also activated by another vitamin A metabolite, all-trans retinoid acid (27). The actions of the nuclear receptors on transcription are mediated by the recruitment of coregulators: coactivators and corepressors. When bound to corepressors such as NCoR (or nuclear receptor corepressor 1) or SMRT (silencing mediator of retinoic and thyroid receptor or NCoR2), the receptors can cause chromatin compaction and repress transcription because corepressors belong to multicomponent complexes that contain histone deacetylases (HDACs) (28). Corepressors play critical and specific roles in nuclear receptor action *in vivo* (29, 30). NCoR is preferentially associated with the TRs, while SMRT shows a preference for RARs (31). Although NCoR/SMRT were first identified by their interaction with TRs and RARs, later studies demonstrated that they also could bind to other numerous transcription factors (32). Conformational changes upon ligand binding lead to the dissociation of corepressors and the recruitment of coactivator complexes, which include proteins with histone acetyl-transferase (HAT) activity such as CBP/p300 or p160 coactivators that allow an open chromatin conformation to facilitate transcriptional activation (24, 25).

Several nuclear receptors, including Esrrb (16, 33, 34), Dax1 (35), the Nr5a family (36–38) and RARs (38–40) have been demonstrated to play essential roles in the acquisition and maintenance of pluripotency of human and mouse cells (41). In addition, the stable repression of maternal Dlk1-Dio3 transcripts acts as a roadblock for the establishment of full pluripotency in iPSCs (42). Dio3 is a deiodinase that regulates thyroid hormone signaling catalyzing the conversion of T4 and T3 to their inactive forms (23). This implies that thyroid hormones could be involved in cellular reprogramming. Accordingly, it has been described that T3 can enhance the reprogramming of somatic human cells (43). Nuclear receptors coactivators and corepressors can also regulate somatic cell reprogramming. Thus, the p160 coactivator NCoA3 is required for iPSC generation (44) and bromodomain inhibition of the coactivator CBP/p300 facilitates efficient reprogramming of human iPSCs, while catalytic inhibition of this coactivator prevents iPSC formation (45). In addition, NCoR and SMRT have been described to cooperate with c-Myc to create an epigenetic barrier to somatic cell reprogramming (46).

The aim of this work has been to define the effect of T3 on reprogramming of mouse embryonic fibroblasts (MEFs) and to explore a possible role of NCoR in this process. Our results show that T3 induces a strong increase of MEFs reprogramming to iPSCs, although its effect is less potent than that induced by 9c-RA. Although there is a strong increase of *TRα* and *TRβ* transcripts during reprogramming, TRβ is the main receptor isoform mediating this effect of T3. iPSCs generated following T3 treatment show increased expression of pluripotency genes, the promoter regions of some of these genes undergo loss of DNA methylation resembling the pattern found in ES cells, and iPSCs were able to form embryoid bodies (EBs) that gave rise to cells of the three germ layers, displaying major hallmarks of pluripotency. Unexpectedly, NCoR appears to be required for the induction of cellular reprogramming, since depletion of the corepressor blocked to a significant extent basal reprogramming, as well as the number of iPSC colonies generated by T3, and also by 9c-RA, inhibiting expression of pluripotency genes.

## 2 Materials and methods

### 2.1 Experimental animals

All animal work was done in compliance with European Union Council guidelines (directive 2010/63/UE) and Spanish regulations (RD 53/2013), with approval of the Ethics Committee of the Consejo Superior de Investigaciones Científicas and by the Comunidad Autónoma de Madrid Review Board (PROEX 053/15). Mice were maintained under a 12:12 light–dark cycle, at a constant temperature of 22°C ± 2°C and *ad libitum* access to food and water. Experiments were carried out with 3 different sets of animals: I) transgenic mice carrying a lentiviral doxycycline-inducible polycistronic cassette encoding the four murine Yamanaka factors *Oct4*, *Sox2*, *Klf4*, and *c-Myc* (3), a kind donation of M. Serrano and María Abad; II) wild-type C57BL/6 mice were used for experiments with retroviral vectors; and III) TRα1^−/−^/TRβ^+/+^ (KOα) mice, TRα1^+/+^/TRβ^−/−^ (KOβ) mice, rederived to a CD1 genetic background, and wild-type TRα1^+/+^/TRβ^+/+^ embryos with the same genetic background (47). KO mice lines were maintained in heterozygosis by crossing heterozygous male mice with heterozygous female mice. For induction of pluripotency experiments, homozygous crosses were used for obtaining WT and KO MEFs. Mice were genotyped (48) and used for the analysis of the contribution of each TR.

### 2.2 Primary cultures and cell lines

Primary cultures of mouse embryonic fibroblasts (MEFs) from the animals described above were obtained from embryos at E13.5 and cultured in standard medium (DMEM supplemented with 10% of FBS and penicillin-streptomycin). All the experiments were performed with MEFs at an early passage (passage 2-4). HEK293T cells used for transfection experiments were cultured in the same medium. Murine ES cell line R1 (ATTC, SCRC-1011), used as a control for stemness, was cultured in iPSC medium: high-glucose DMEM supplemented with 15% serum replacement (KSR, Invitrogen), leukemia inhibitory factor (LIF;1,000 U/ml, Millipore), non-essential amino acids, glutamax, penicillin-streptomycin and β-mercaptoethanol. Cultures were routinely tested for mycoplasma and were always negative.

### 2.3 MEFs reprogramming

For OSKM transgenic mice-derived MEFs, induction of reprogramming was initiated with iPSC medium supplemented with doxycycline (1µg/ml). Reprogramming of MEFs using exogenous Yamanaka factors was performed as previously described (49), using the retroviral plasmids pMXs-Oct4, pMXs-Sox2, pMXs-Klf4 or pMXs-cMyc (obtained from Addgene). Briefly, HEK293T cells were transfected with the plasmid and packaging vectors using Fugene 6 (Promega). Viral supernatants were collected twice a day on two consecutive days starting 24 h after transfection and were used to infect MEFs, adding polybrene (8 µg/ml). MEFs were treated with the supernatants containing the four factors (OSKM) or with different combinations of three factors (OSK, OSM, SKM), or two factors and cultured in iPSC medium starting 24h after the last treatment. For hormonal treatment, iPSC medium was supplemented with 10nM T3, 1µM 9c-RA, or 10nM GC-1 (all from Sigma). The reprogramming medium was replaced every other day. For quantification of retroviral transduction efficiency, parallel infections containing all the retroviruses used for reprogramming plus a GFP retroviral supernatant (pBABE-PURO-GFP plasmid) were performed, followed by FACS analysis at day 3 post-infection. The total number of AP^+^ colonies was counted at different time points after staining the plates for AP activity (BCIP/NBT color development substrate kit, Promega). Cell proliferation was analyzed in 96-well plates (40,000 cells/well) using a colorimetric MTT kit (Calbiochem) following manufacturer’s recommendations at different days of reprogramming in the presence and absence of T3 or 9c-RA.

### 2.4 NCoR silencing

Lentiviral plasmids for shRNA-mediated *NCoR* gene silencing were purchased from Sigma and the better performing one (TRCN0000318009) was used in the assays. TRCN0000096477 plasmid transduction resulted in negligible silencing as compared to the empty vector (pLKO.1-puro vector) or no vector, and was used as a control shRNA. Self-inactivating replication incompetent viral particles were produced in HEK293T packaging cells by co-transfection with packaging plasmids using Fugene 6. Twenty-four hours after transduction, the protocol for reprogramming was started by incubation with doxycycline in the presence and absence of T3 or 9c-RA in iPSC medium. For silencing efficiency tests, parallel untreated plates were harvested after 3 days and RNA and/or protein were extracted. *NCoR* mRNA silencing was also tested at the end of the experiments.

### 2.5 Colony expansion

Individual colonies were picked, disaggregated with trypsin, seeded on mitomycin-inactivated MEF feeders in iPSC medium and cultured without doxycycline in the absence or presence of T3, first to 24-wells plates and then to P60 plates. Expanded colonies were used for further analysis of gene expression and DNA methylation after removal of MEF feeders by plating for 30 min.

### 2.6 EBs formation

The hanging-droplet culture method was used for generating EBs. Briefly, single cells from expanded individual colonies were harvested by trypsinization and diluted in LIF-free iPSC medium, to allow for spontaneous differentiation in the absence and presence of T3. Hanging droplets of 20 µl (5,000 cells per droplet) were suspended on the underside of P100 Petri dishes lids. After 3 days, each droplet was transferred to a non-adherent round-bottom P96 well for 5 days and then to a P24 well for 7 or 18 days, when they were harvested for histological staining.

### 2.7 Western blot

Cell extracts were prepared using RIPA buffer, resolved on 12% acrylamide gels and transferred to a nitrocellulose membrane. The membrane was cut and hybridized with antibodies against NCoR (270kDa), and lamin B (67kDa) used as a loading control.

### 2.8 Immunodetection

For immunofluorescence cells were grown in 24-well plates, fixed with 4% paraformaldehyde, washed with PBS, and blocked with 4% BSA. For whole mount immunofluorescence EBs were seeded on 8-wells chambered coverslips (IBIDI, 80826), fixed, blocked and permeabilized as described in Martí et al. (50). Cells or EBs were incubated overnight with the corresponding primary antibody, washed with PBS and incubated with secondary antibodies. Samples were also counterstained with DAPI (Invitrogen, 21490). Slides were mounted with ProLong (Molecular Probes) and analyzed with an Espectral Leica TCS SP5 confocal microscope (40×/1.25–0.75 oil). Maximum-intensity projections of the image stacks were then generated using Software LAS-AF. A subset of EBs were fixed, paraffined and cut on 5 µm sections on Superfrost slices for standard Hematoxylin&Eosin staining or immunohistochemistry. Images were analyzed by light microscopy (Zeiss Axiophot microscope with DP70 Olympus digital camera). The primary and secondary antibodies used in this study are listed in **Supplementary Table 1**.

### 2.9 Quantitative real time PCR

Total RNA was extracted from whole wells with Trizol (Life Technologies) or with ReliaPrep™ RNA Cell Miniprep System (Promega). Samples were treated with DNase I before reverse transcription using iScrip cDNA Synthesis Kit (BioRad), according to the manufacturer’s protocols. Quantitative real-time PCR was performed using an Mx3005P (Stratagene) and Power Sybr Green (Applied Biosystems). Calculation was made using the ΔΔCt method using Gus1 mRNA as an internal control. **Supplementary Table 2** shows name and sequence for the used primers.

### 2.10 DNA methylation analysis by bisulfite sequencing

Genomic DNA isolation was performed according to a standard phenol/chloroform/isoamyl alcohol extraction protocol after proteinase K digestion. Bisulfite conversion of isolated DNA was performed in accordance with the EZ DNA Methylation Kit (Zymo Research) following the manufacturer’s instructions. After PCR amplification, pyrosequencing was performed using PyroMark Q24 reagents and a vacuum prep workstation, equipment and software (Qiagen) as described (51). The PyroMark Assay Design tool (v. 2.0.01.15) was used to design the pyrosequencing oligonucleotides shown in **Supplementary Table 3** for the amplification and pyrosequencing of the target genes.

### 2.11 Statistical analysis

Two-tailed Student’s t-tests were used for comparisons between two groups. One-way ANOVA or Two-way ANOVA with *post-hoc Tukey* test for multiple comparisons were used to compare data from at least three different groups, and linear regression analysis was used to analyze correlation between XY pairs. The results are always expressed as means ± SEM. P values <0.05 were considered statistically significant. The significance of the statistical test in the Figures is shown as *p < 0.05, **p < 0.01, and ***p < 0.001. Statistics were performed with GraphPad Prism 7.0 software.

## 3 Results

### 3.1 T3 enhances reprogramming of MEFs to iPSCs

**Figure 1:**
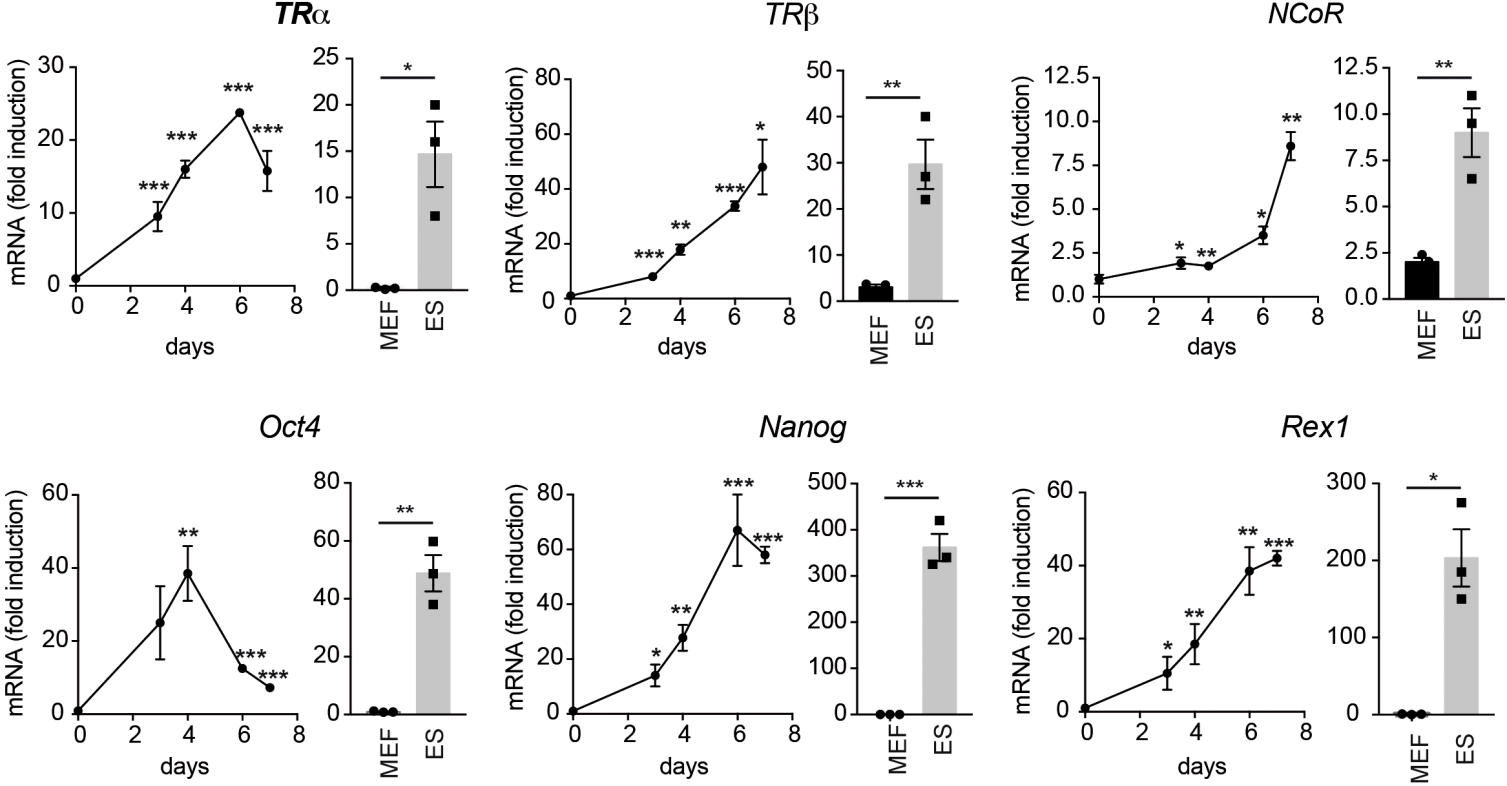
Reprogramming of OSKM-inducible MEFs increases the expression of T3-related transcripts and stemness markers. The main graphs show the mRNA levels of *TR*α, *TR*β and *NCoR* (upper panels) and of the stemness factors *Oct4*, *Nanog* and *Rex1* (lower panels) during reprogramming of OSKM-inducible MEFs (from day 0 to day 7 of doxycycline treatment), n=3. Data are expressed relative to the values obtained at day 0 and asterisks indicate the existence of significant differences with respect to the values obtained at time 0. * p<0.05; ** p<0.01; *** p< 0.001. For comparison, beside each graph, the relative transcript levels in non-reprogramed MEFs and in ES cells cultured in parallel and t-tests between both groups is also shown. Note that the Y axis of the panels corresponding to ES cells is in some cases different.

**Figure 2:**
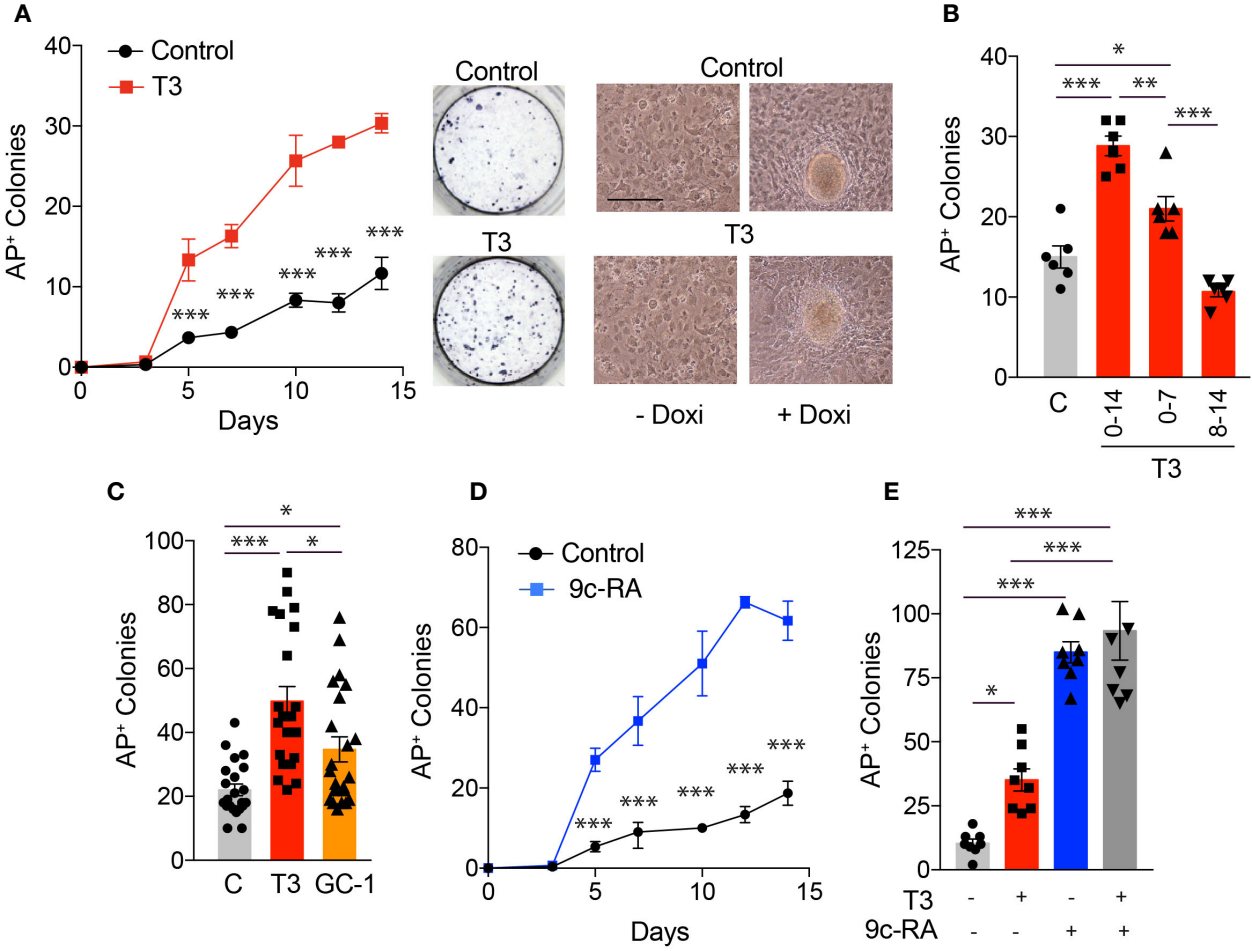
T3 treatment increases the number of iPSC colonies produced by reprogramming OSKM-inducible MEFs. **(A)** Time course for the appearance of alkaline phosphatase positive (AP^+^) iPSC colonies in presence or absence of T3 (from day 0 to day 14 of induction with doxycycline), n=3. Asterisks indicate statistically significant differences between control and T3-treated cells were obtained by two-way ANOVA. Representative photographs of AP^+^ stained colonies at day 12 of induction are shown. Left images show *in vivo* photographs of MEFs cultures after 1 day (left) and 10 days (right) of incubation with doxycycline in control and T3-treated MEFs. Scale bar 100 µm. **(B)** Number of AP^+^ colonies in MEFs treated with doxycycline for 14 days in the absence of T3 (C) or in the presence of T3 from days 0 to 14; 0 to 7 or 8 to 14. **(C)** Number of AP^+^ iPSC colonies at day 12 of incubation with doxycycline in MEFS with no treatment, or treated with T3 or with GC-1, n=22. Statistical comparisons by one-way ANOVA. **(D)** Number of AP^+^ colonies obtained in the presence or absence of 9c-RA (from day 0 to day 14 of induction with doxycycline), n=3. Significant differences between control and 9c-RA treated cells obtained by two-way ANOVA analysis are shown by asterisks. **(E)** Number of AP^+^ iPSC colonies at day 11 of incubation with doxycycline in MEFs with no treatment, or treated with T3, 9c-RA or both, n=8. In all cases: *p<0.05, **p<0.01, ***p<0.001.

To analyze the role of TRs in cellular reprogramming we began by measuring the expression levels of *TRα* and *TRβ* transcripts, as well as of *NCoR*, in MEFs, in a line of mouse ES cells and in MEFs from transgenic mice carrying a lentiviral doxycycline-inducible polycistronic cassette encoding the four murine factors *Oct4, Sox2, Klf4* and *c-Myc* (OSKM) after 0-7 days of incubation with doxycycline (**Figure 1**). These MEFs can be efficiently reprogrammed to generate iPSCs in the presence of doxycycline (3). The levels of the three transcripts were much lower in MEFs than in ES cells and were gradually induced by incubation of the transgenic MEFs with doxycycline, reaching values similar to those observed in ES cells. This increase was concomitant with an increased expression of the stemness markers *Oct4, Nanog* and *Rex1* that, as expected, were essentially undetectable in MEFs and displayed a very high expression in ES cells. These results suggest a role for TRs in the induction of iPSCs from MEFs. Therefore, we next analyzed the effect of T3 on the kinetics of MEFs reprogramming by determining the appearance of AP positive (AP^+^) colonies. As shown in **Figure 2A**, treatment with the hormone strongly enhanced reprogramming efficiency, although it did not appear to shorten the reprogramming course, as colonies emerged after 5 days of doxycycline incubation both in control and T3-treated cells. A significant increase in the number of colonies was already observed at day 5 and was maintained up to 14 days of treatment, when cultures start to detach from the plate. Treatment with T3 during the first 7 days also increased reprogramming, although less strongly than when T3 was present during the whole period, while the hormone was ineffective when treatment started at day 7 (**Figure 2B**). Thus, T3 appears to be required at the initial stages to enhance reprogramming. In addition, the TRβ-specific ligand GC-1 (sobetirome), also increased the number of AP^+^ colonies, although its effect was somewhat lower than that of T3, which binds both to TRα and TRβ (**Figure 2C**). 9c-RA also increased significantly the efficiency of MEFs reprogramming from days 5 to day 14 of doxycycline treatment (**Figure 2D**). At day 11 the effect of T3 on MEFs reprograming was less marked than that induced by 9c-RA (3.4 and 8.1-fold, respectively) and the combination of both ligands did not induce a further increase (**Figure 2E**).

**Figure 3:**
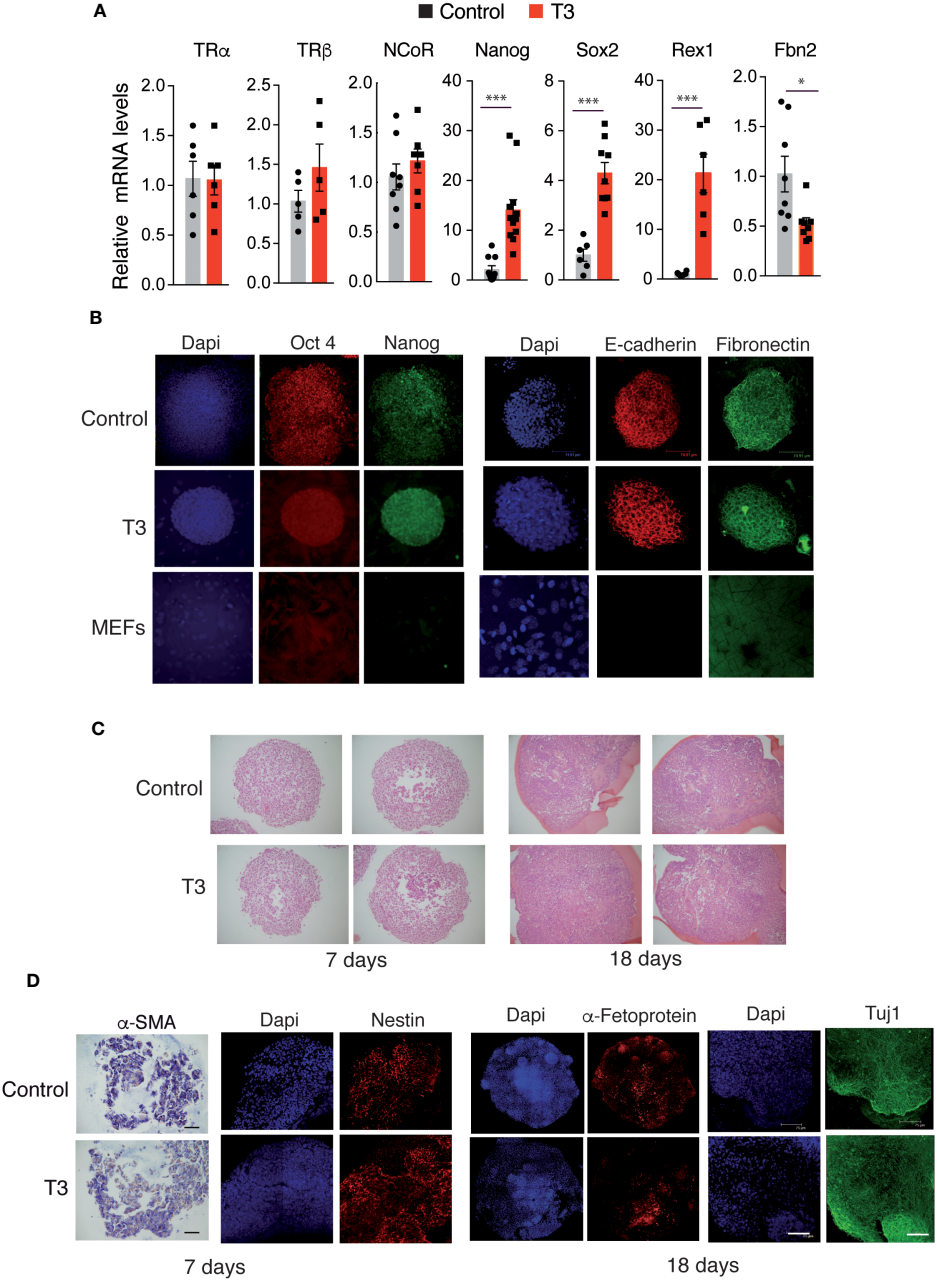
T3 treatment enhances OSKM-inducible MEFs reprogramming features. **(A)** Transcript levels of *TRα*, *TRβ*, *NCoR*, *Nanog*, *Sox2*, *Rex1* and *Fbn2* at day 11 of incubation with doxycycline in control and T3-treated MEFs, n=8. Data are expressed relative to the values obtained in the control untreated cells. Statistical comparisons by two-tailed *t* test: *p<0.05, **p<0.01, ***p<0.001. **(B)** Detection of the stemness markers Oct4 and Nanog and of the epithelial markers E-cadherin and Fibronectin by immunofluorescence in non-treated and T3-treated iPSC colonies after 5 days of incubation with doxycycline, and in non-induced MEFs. Scale bar 75 µm. **(C)** Sections of embryoid bodies (EBs) obtained from trypsinized iPSC colonies after 7 or 18 days of formation stained with Hematoxylin&Eosin. Scale bar 100 µm. **(D)** Whole EBs stained for markers of the three germ layer cell types (α-SMA by immunohistochemistry, and immunofluorescence staining for Nestin, Tuj1, and α-Fetoprotein). α-SMA, smooth muscle actin. Scale bar 75 µm.

To study the effect of T3 on gene expression during MEFs reprograming, we analyzed by real time quantitative PCR transcript levels of the *TRs* and *NCoR*, of several pluripotency markers and of the fibroblast marker *Fbn2* after 11 days of doxycycline treatment. The hormone did not alter *TR*s or *NCoR* transcripts, but induced a significant increase of *Nanog*, *Sox2* and *Rex1* mRNA levels, while down-regulating *Fbn2* mRNA, in agreement with the enhanced number of reprogrammed colonies in the cultures (**Figure 3A**).

To further characterize the iPSC colonies, the expression of the key pluripotency markers Oct4 and Nanog was analyzed by immunofluorescence. Colonies generated in the absence and presence of T3 were stained, while non-reprogramed MEFs did not express these ES cell markers. In addition, reprogrammed cells in the colonies, but not isolated non-reprogrammed MEFs, had an epithelial phenotype, as demonstrated by an abundant expression of E-cadherin, which is essential for the maintenance of pluripotency of mouse ES cells and is associated with the reprogramming of MEFs to iPSCs (52) and also expressed fibronectin, required for the self-renewal of ES cells (53) (**Figure 3B**).

Once iPSCs activate pluripotency genes they no longer require exogenous expression of the Yamanaka factors to sustain a pluripotent state and can form EBs under non-adherent conditions (2). Trypsinized iPSC colonies were able to generate EBs both in the absence and presence of T3 without further doxycycline treatment (**Figure 3C**). Moreover, EBs differentiated into the three germ layer cell types, as shown by the detection of the mesodermal marker α-smooth muscle actin (α-SMA) by immunohistochemistry, and by immunofluorescence staining with Nestin and Tuj1 (ectodermal markers), as well as α-Fetoprotein (endodermal marker) (**Figure 3D**).

**Figure 4:**
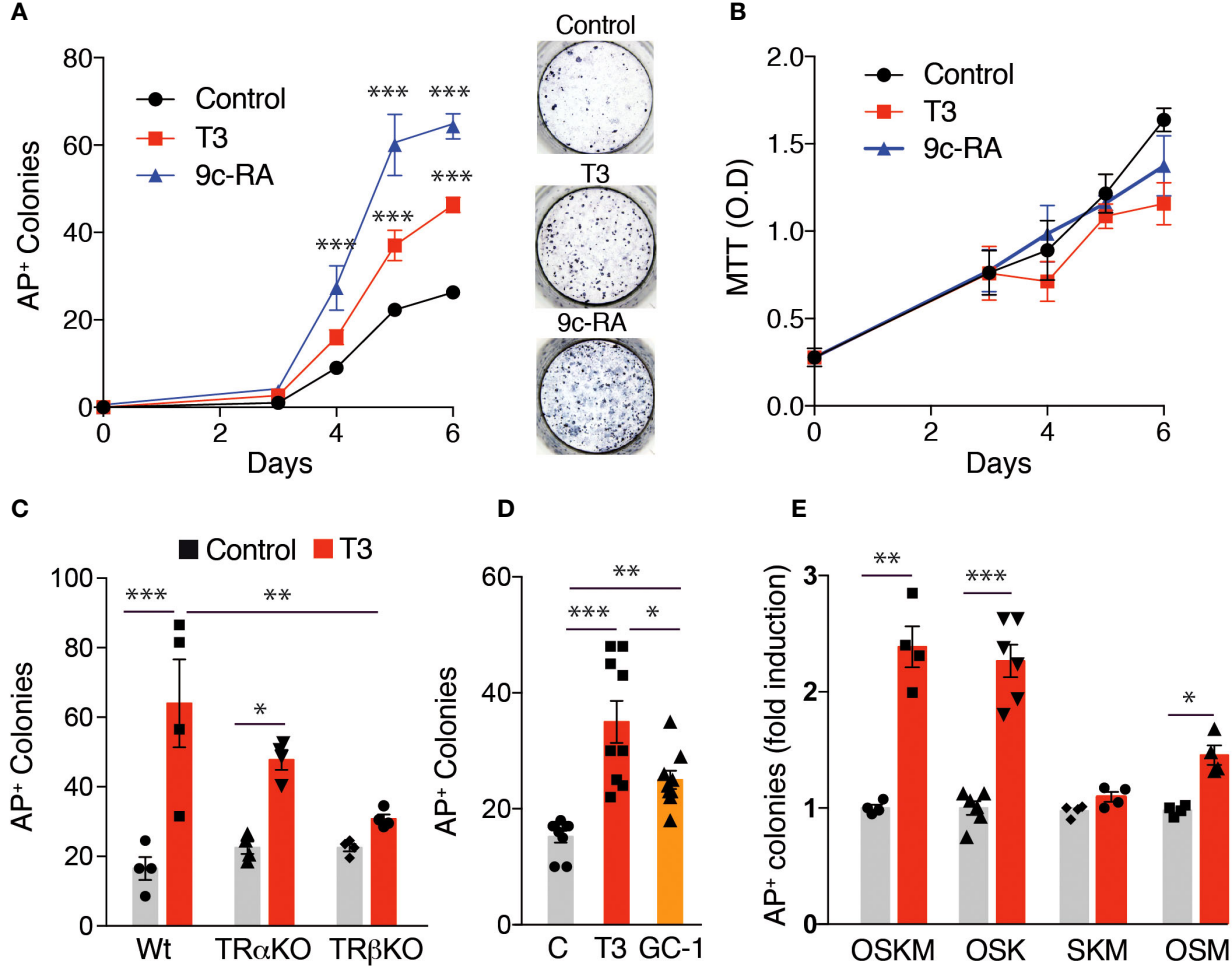
Effect of T3 or 9c-RA treatment on MEFs reprogrammed with the Yamanaka factors. **(A)** Time course for the appearance of alkaline phosphatase positive (AP^+^) iPSC colonies in non-treated, T3-treated, or 9c-RA-treated MEFs transduced with retroviral vectors encoding the OSKM factors (from day 0 to day 6 of treatment), n=3. Asterisks indicate statistically significant differences between control and 9c-RA or T3-treated cells obtained by two-way ANOVA. Representative photographs of AP^+^ stained colonies at day 12 after transduction. **(B)** MTT analysis of cell proliferation in non-treated, T3-treated, and 9c-RA-treated MEFs transduced with the OSKM factors (from day 0 to day 6 of treatment), n=5. **(C)** Number of AP^+^ iPSC colonies at day 12 after transduction with the OSKM factors in untreated and T3-treated MEFs derived from WT, TRα KO or TRβ KO mice, n=4. Statistically significant differences analyzed by two-way ANOVA are shown with asterisks. **(D)** Number of AP^+^ iPSC colonies at day 12 after transduction with OSKM in MEFS with no treatment, or treated with T3 or with GC-1, n=9. One-way ANOVA was used to find statistically significant differences. **(E)** Induction by T3 of the number of AP^+^ iPSC colonies in MEFs at day 12 after transduction with OSKM, OSK, SKM, or OSM factors, n=6. Statistical comparisons by two-way ANOVA: *p<0.05, **p<0.01, ***p<0.001.

To further analyze the role of T3 on iPSC generation, we used an alternative method of MEFs reprograming by retroviral infection with the individual Yamanaka factors. The effect of T3 on MEFs transduced with OSKM factors was essentially identical to that obtained with the transgenic MEFs, increasing the number of AP^+^ colonies from day 4, and again a stronger effect of 9c-RA was observed (**Figure 4A**). The reprogramming process requires initial cell proliferation (54, 55). However, the increased reprogramming efficiency by T3 or 9c-RA-treated MEFs was not due to cellular proliferative activity, because 9c-RA did not alter cell growth and T3 even had a weak negative effect (**Figure 4B**). This reprogramming protocol allowed us to distinguish between the role of TRα and TRβ on the effect of T3 by using MEFs from genetically modified mice lacking the receptors. While the hormone caused a significant increase in the number of AP^+^ colonies generated by wild-type and TRα knockout MEFs, its effect was not significant when the cells lacked TRβ (**Figure 4C**). Similar to the results shown in **Figure 2C**, the TRβ specific ligand GC-1 also induced a significant enhancement of the number of reprogrammed colonies after transduction with the retroviral OSKM factors (**Figure 4D**). These results, again suggest that the TRβ isoform plays an important role in the increased T3-dependent efficiency of MEFs reprogramming. We next investigated whether in addition to enhancing reprogramming efficiency T3 could replace the core reprogramming factors. c-Myc has already been demonstrated to be dispensable for reprogramming (56), and T3 was equally effective in increasing the number of AP^+^ colonies in MEFs transduced with OSKM and OSK. However, T3 was ineffective in inducing colony formation in MEFs transduced with SKM, while having a weak effect in cells transduced with OSM (**Figure 4E**). In addition, no colonies were observed in the absence or presence of T3 in cells transduced with only two factors (OS, SK or SM) (**Figure S1**). Therefore, T3 could not replace Oct4, and could only very partially replace Klf4 when c-Myc is present, in agreement with the finding that the hormone can induce expression of Klf transcription factors (57, 58).

### 3.2 Depletion of NCoR reduces MEFs reprogramming even in the presence of T3

**Figure 5:**
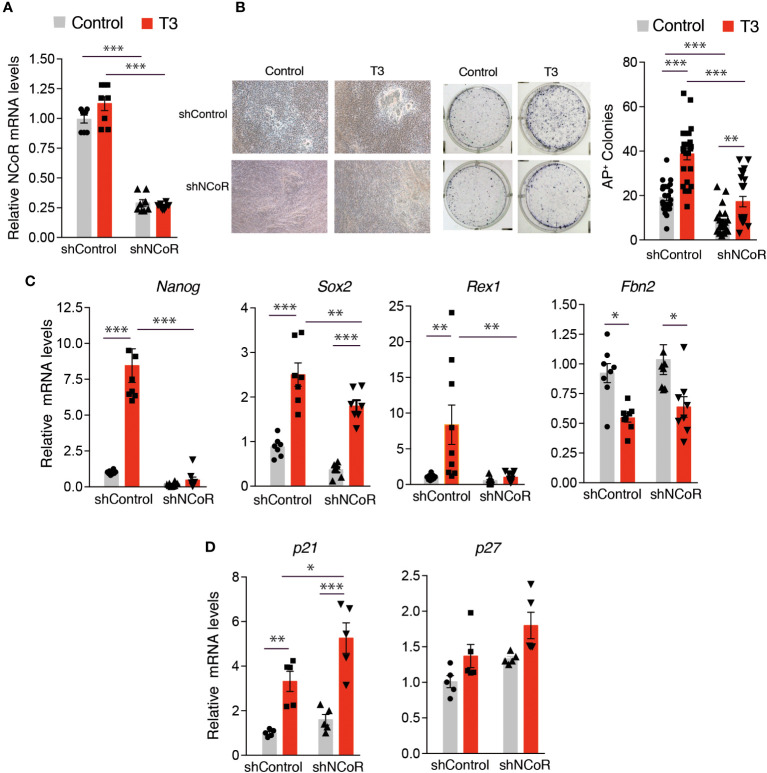
NCoR corepressor plays a critical role in MEFs reprogramming to iPSCs. **(A)** *NCoR* mRNA levels after transduction with shControl or shNCoR of control and T3-treated OSKM-inducible MEFs (after 12 days of doxycycline treatment), n=8. **(B)** Representative photographs and quantification of AP^+^ stained colonies at day 12 of induction, n=24. **(C)** Transcript levels of *Nanog*, *Sox2*, *Rex1* and *Fbn2* at day 11 of incubation with doxycycline in control and T3-treated MEFs after transduction with shControl or shNCoR, n=8. **(D)** Transcript levels of the cyclin-dependent kinase inhibitors *p21* and *p27* under the same conditions, n=5. Transcript levels are always expressed relative to the values obtained in untreated cells transduced with shControl. Statistical comparisons were performed by two-way ANOVA and are indicated as: *p<0.05, **p<0.01, ***p<0.001.

As NCoR was found to be up-regulated together with TRα and TRβ during iPSC generation (**Figure 1**), we next tested the possibility that the corepressor may also be important for the induction of reprogramming by T3. To determine how NCoR affects reprogramming, we used a specific shRNA to knockdown the corepressor in the transgenic MEFs carrying OSKM and observed a knockdown efficiency >70% after 72h of incubation with doxycycline both at the level of mRNA and protein (**Figure S2A**). At this time, control and NCoR knockdown MEFs showed no difference in cell proliferation (**Figure S2B**). *NCoR* mRNA depletion was maintained during reprogramming both in the absence and presence of T3, being reduced by 75% after 12 days of doxycycline treatment (**Figure 5A**). Depletion of the corepressor caused a drastic reduction in reprogramming efficiency, as judged by the significantly lower number of AP^+^ colonies generated, reversing to a significant extent T3-induced reprogramming (**Figure 5B**). In parallel with the reduced iPSC generation, induction by T3 of transcripts for the pluripotency markers *Nanog*, *Sox2* and *Rex1* was dramatically reduced in NCoR-depleted cells. In contrast, no significant differences in *Fbn2* mRNA levels were observed (**Figure 5C**). We next asked whether NCoR knockdown might inhibit reprogramming by inducing the cyclin kinase inhibitors p21 and p27, a known impediment for acquisition of pluripotency (49, 59, 60). Despite increasing iPSC generation, incubation with T3 led to a significant increase of *p21* transcripts compared with the untreated cells transduced with control shRNA and this increase was more marked in NCoR-depleted cells. The levels of *p27* transcripts showed a similar trend, but differences were not statistically significant (**Figure 5D**).

**Figure 6:**
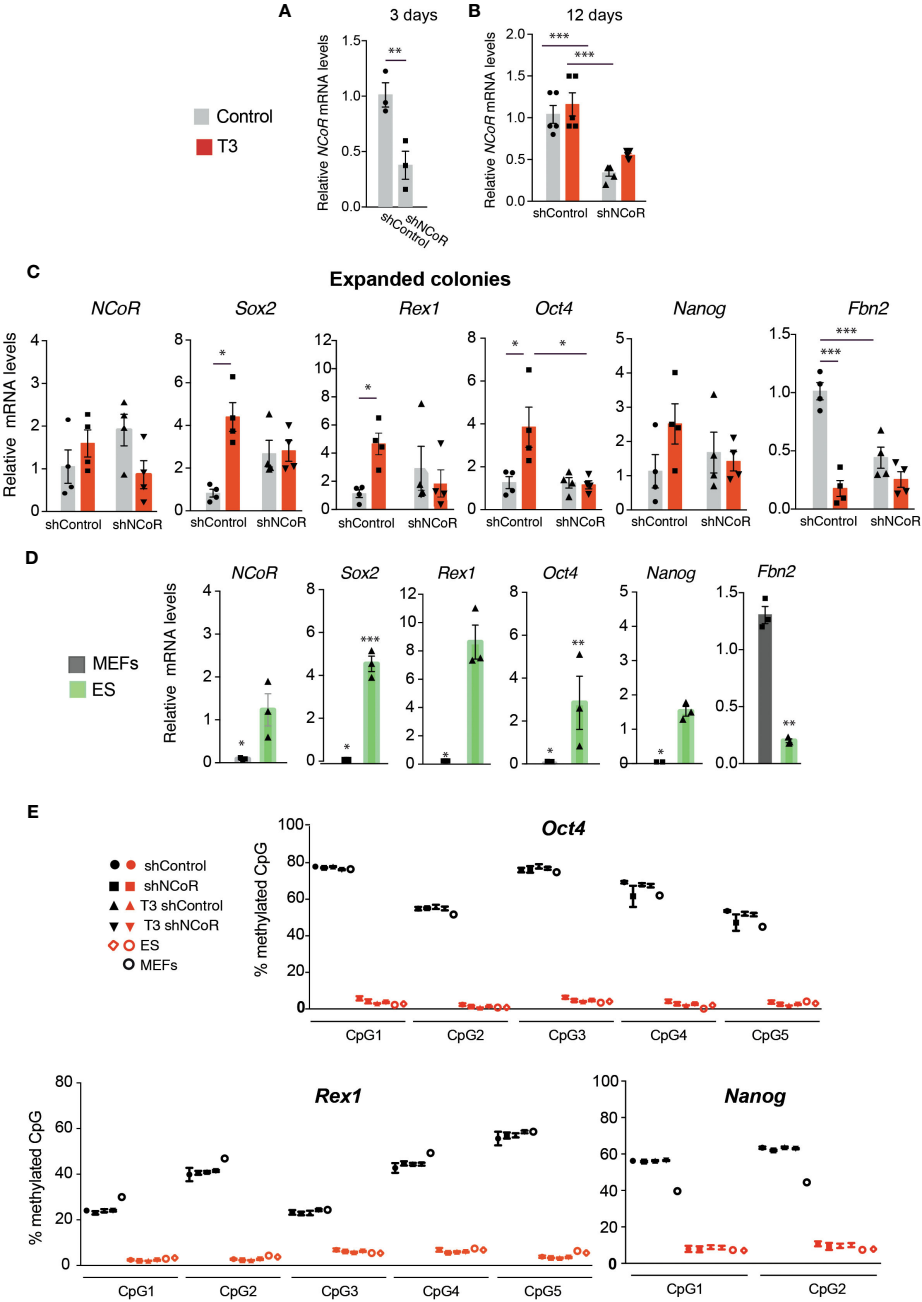
NCoR silencing interferes with MEFs reprogramming to iPSCs. **(A)** Levels of *NCoR* mRNA 3 days after transduction of OSKM-inducible MEFs with shControl or shNCoR and doxycycline treatment, n=5. **(B)** *NCoR* transcripts in the same experiment at day 12 of incubation with doxycycline in control and T3-treated MEFs after transduction of shControl or shNCoR, n=5. **(C)** iPSCs from the colonies of this experiment were picked after 13 days of treatment and expanded in the absence and presence of T3. Expression levels of *NCoR*, *Sox2*, *Rex1*, *Oct4*, *Nanog* and *Fbn2* mRNA were measured in the iPSCs from the expanded colonies, n=4. Transcript levels are always expressed relative to the values obtained in untreated cells transduced with shControl. Statistical comparisons were performed by two-way ANOVA: *p<0.05, **p<0.01, ***p<0.001. **(D)** mRNA levels of the same genes were measured in the same assay in MEFs and ES cells cultured in parallel for 6 days, n=3. Results are expressed relative to the values obtained in the untreated expanded iPSCs transduced with shControl and asteriks denote significant differences with respect to that group. **(E)** The percentage of DNA methylation of the individual CpG islands present in the promoters of the pluripotency genes *Rex1*, *Oct4*, and *Nanog* in the expanded iPSC colonies under the same conditions as in **(C)** is shown in red symbols, n=6. CpG islands methylation in non-reprogrammed MEFs after transduction with shControl or shNCoR in the absence and presence of T3 after 6 days of incubation without doxycycline is shown with black symbols, n=6. The DNA methylation pattern of control MEFs and in two different ES cell cultures is also illustrated.

The finding that efficiency of NCoR depletion was not total opened the possibility that the cells that escape knockdown could have an advantage for converting into iPSCs. Thus, we next measured initial knockdown of *NCoR* mRNA levels in the cultures 3 days after transduction with shNCoR, 12 days after transduction and doxycycline treatment in the absence and presence of T3 and in iPSC colonies picked 13 days after doxycycline and/or T3 treatment and expanded without doxycycline. As expected and confirming the results shown in **Figure S2**, shNCoR reduced very significantly, and at to a similar extent, initial corepressor transcripts (**Figure 6A**) and transcripts in the cultures after 12 days of doxycycline composed by a mixed population of non-reprogrammed and reprogrammed MEFs (**Figure 6B**). However, *NCoR* mRNA levels showed a higher variability in the iPSCs expanded from the isolated colonies and no statistically significant differences between cells transduced with shControl and shNCoR were found (**Figure 6C**). We also analyzed expression of pluripotency genes in these cells (**Figure 6C**), which were compared in the same assay with those of ES cells and non-reprogrammed MEFs (**Figure 6D**). T3 treatment was still able to increase pluripotency gene expression in the iPSCs, while repressing very strongly *Fbn2* gene expression Interestingly, *Sox2*, *Rex1*, *Oct4* and *Nanog* transcripts showed levels comparable to those found in ES cells, suggesting that the majority of cells could have undergone complete reprogramming (**Figures 6C, D**). The pattern of gene expression observed suggested that *NCoR* transcripts could correlate with those of the analyzed genes. Indeed, when *NCoR* mRNA levels where plotted against the mRNA levels of the pluripotency genes, independently of the experimental group, a statistically significant positive correlation was found with all of them, while *Fbn2* mRNA showed an opposite trend (**Figure S3**).

Fully reprogrammed iPSCs are characterized by displaying notable DNA demethylation of pluripotency genes, which seems to be crucial for faithful reprogramming (19). Therefore, we next analyzed the DNA methylation pattern of several ES marker genes in the expanded iPSC colonies. Analysis of the promoters of the *Rex1*, *Oct4* and *Nanog* genes showed a fully demethylated state of their CpG islands in the iPSCs, independently of previous transduction with shRNA and the T3 treatment, resembling the DNA methylation pattern found in ES cells. As expected, these promoters showed strong DNA methylation marks in MEFs (**Figure 6E**). DNA methylation analysis also revealed that the CpG islands of the *Sox2* and *Fbn2* promoters were largely unmethylated in the reprogrammed cells, as well as in ES cells and MEFs (**Figure S4**).

### 3.3 Influence of NCoR depletion on MEFs reprogramming by 9c-RA

**Figure 7:**
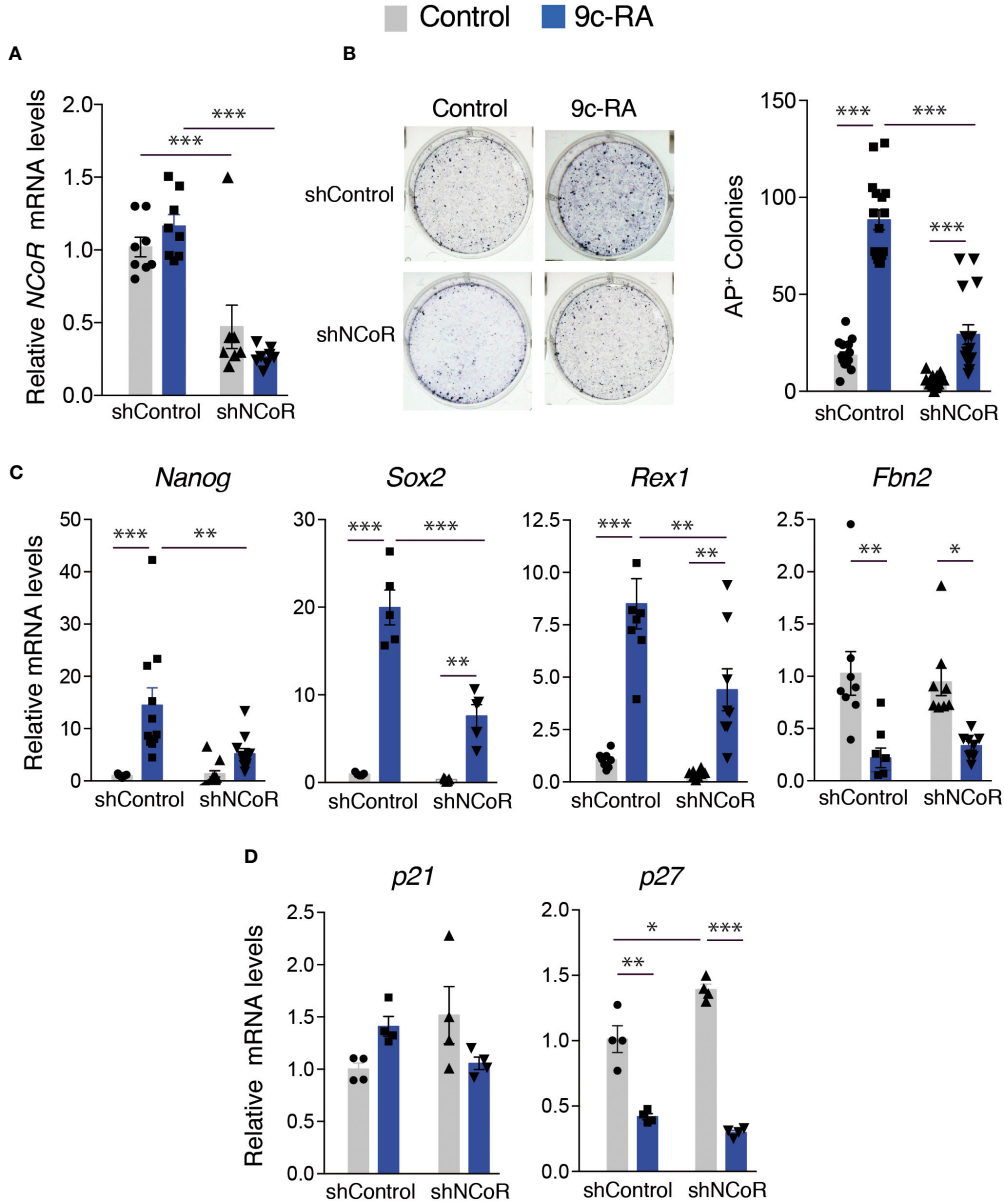
NCoR is involved in the induction of MEFs reprogramming to iPSCs by 9c-RA. **(A)** *NCoR* mRNA levels after transduction with shControl or shNCoR of control untreated and 9c-RA-treated OSKM-inducible MEFs (after 12 days of doxycycline treatment), n=9. **(B)** Representative photographs and quantification of AP^+^ stained colonies at day 12 of induction in control and 9c-RA-treated MEFs after transduction with shControl or shNCoR, n=18. **(C)** Transcript levels of *Nanog*, *Sox2*, *Rex1* and *Fbn2* at day 12 of incubation with doxycycline in control and 9c-RA-treated MEFs transduced with shControl or shNCoR, n=9. **(D)** Transcript levels of the cyclin-dependent kinase inhibitors *p21* and *p27* at day 12 of incubation with doxycycline in control and 9c-RA-treated MEFs after transduction with shControl or shNCoR, n=4. Transcripts levels are always expressed relative to the values obtained in untreated cells transduced with shControl. Statistical comparisons were performed by two-way ANOVA: *p<0.05, **p<0.01, ***p<0.001.

We next analyzed the role of NCoR on stimulation of iPSCs generation by 9c-RA. For this purpose, we checked *NCoR* transcripts, as well as the appearance of AP^+^ colonies in the transgenic MEFs after 12 days of incubation with doxycycline in the presence and absence of 9c-RA. NCoR was efficiently depleted both in untreated and 9c-RA treated cells (**Figure 7A**) and, similar to the results obtained with T3, under these conditions a strong reversion of the effect of the rexinoid in reprogramming was observed (**Figure 7B**). Treatment with 9c-RA also caused a strong induction of the expression of the pluripotency genes *Nanog, Sox2* and *Rex1* and this response was also significantly blunted in the NCoR-depleted cells, in agreement with the reduced reprogramming efficiency. Again, *Fbn2* mRNA levels were reduced by 9c-RA, and NCoR knockdown did not elicit major changes (**Figure 7C**). In contrast with T3 that increased *p21* expression, 9c-RA had no significant effects, and opposite to T3 was able to repress significantly *p27* transcripts, which might facilitate reprogramming (**Figure 7D**).

## 4 Discussion

We show here that T3, a hormone with an important role in cell growth and differentiation (61), also enhances the reprogramming of somatic MEFs to pluripotent stem cells, extending findings previously obtained with human cells (43). The finding that T3 enhances reprogramming in both mouse and human cells reinforces the importance of the hormone in induction of pluripotency. Our results also indicate that TRβ plays an important role in mediating enhanced reprogramming, as shown by the finding that the TRβ-specific ligand GC-1 is almost as strong as T3, which binds both TRα and TRβ, and that T3 causes a more potent increase of reprogramming in TRα- than in TRβ-knockout MEFs. In the experiments we have used a dose of T3 and GC-1 sufficient to fully occupy the receptors, and considering the TRs dissociation constant most likely lower doses of hormone would be able to induce reprogramming. In addition, the other thyroid hormone T4 has a 10-fold less affinity than T3 for the receptor, but can be converted to T3 by the action of intracellular deiodinases (23, 25) and therefore, at higher concentrations than T3, most likely T4 could also enhance MEFs reprogramming.

Induction of MEFs reprogramming by T3 is not as marked as that observed with 9c-RA. This ligand has been shown to bind both RARs and RXRs, and its effect could be at least partially mediated by RARs, which are well-known inducers of somatic cell reprogramming demonstrated by embryoid bodies generation and by formation of teratomas and chimeric mice (37–40). In particular cellular environments RXR can act as a “nonsilent” partner of TRs, allowing stimulation of gene expression by RXR agonists (62). However, the induction of iPSC generation by T3 and 9c-RA was not additive, suggesting that 9c-RA does not act through the TRβ/RXR heterodimer. However, we cannot dismiss the possibility that 9c-RA, besides binding to RARs, could act through other/s so called permissive heterodimers with other nuclear receptors, which can be indistinctly activated by ligands of either RXR or its partner receptor and are synergistically activated in the presence of both ligands (24).

Several nuclear receptors have been demonstrated to play essential roles in cellular reprogramming (41). When coexpressed with RARγ, Nr5a2 significantly accelerates the reprogramming process by the Yamanaka factors (38). Additionally, Nr5a2 and Nr5a1 can replace the core pluripotency factor Oct4 possibly due to their ability to activate Oct4 gene expression (36). This is not the case with T3, which does not increase appreciably the reprogramming kinetics and that cannot substitute Oct4. Although the differentiation potency of the AP^+^ colonies generated by OSM has not been tested, our results suggest that T3 to a limited extent might bypass the need for exogenous Klf4 in reprogramming. The Klf family of transcription factors induce expression of key pluripotency genes, such as *Nanog*, and other Klfs could play a redundant role with Klf4 in the self-renewal of ES cells (63). At this respect, *Klf9* is a well-known target gene of the thyroid hormone in different cell types (47, 57, 64) and T3 has been recently shown to upregulate Klf4 and Klf9 in human iPSCs and in ES cells, where it enhances cell survival and pluripotency (58). On the other hand, T3 can increase the number of AP^+^ colonies in the absence of c-Myc. This is not surprising since Oct4, Klf4 and Sox2 cooperatively suppress expression of somatic genes and induce ES cell-related genes, resulting in the establishment of a self-sustaining pluripotency network (7, 65), while c-Myc is dispensable for iPSC generation, but increases reprogramming presumably by stimulating cell proliferation and inducing a metabolic switch from an oxidative to a glycolytic state that is typical of pluripotent cells (15, 66).

It has been previously shown that increased cell proliferation promotes MEFs reprogramming (54, 55), while increased amounts of cycling-dependent kinase inhibitors (CKIs) induce cell senescence and decrease cell proliferation, thus reducing the efficiency of iPSC generation (49, 59). On the other hand, it has been described that promotion of reprogramming of human dermal fibroblasts by T3 is associated with increased growth rate during the first days (43). Strikingly, increased reprogramming in T3-treated MEFs occurred under conditions of markedly increased *p21* gene expression and reduced proliferation rates. This is agreement with our previous results demonstrating that the thyroid hormones can induce premature senescence in MEFs and *in vivo* (67). Therefore, T3 is able to bypass the inhibitory effect of CKIs and cell senescence, which oppose generation of pluripotent stem cells. Furthermore, induction of cellular senescence by T3 is mediated by TRβ, and not by TRα, and therefore the same receptor isoform might increase both cellular senescence and reprogramming efficiency by the Yamanaka factors. In contrast with T3, 9c-RA does not affect *p21* gene expression and significantly reduces *p27* gene expression, also a roadblock for cellular reprogramming (60). This could be one of the reasons underlying the stronger effect of the rexinoid on MEFs reprogramming and suggests that, most likely, CKI depletion could further enhance the extent of T3-dependent iPSC generation.

Before activating pluripotency genes, MEFs undergo a mesenchymal to epithelial transition characterized by the silencing of mesenchymal genes and the acquisition of epithelial features (11, 12). Previous studies from our laboratory have demonstrated an important role for TRs in different cell types, as well as in cancer and somatic stem cells, through regulation of the activity of signaling pathways such as BMP, Wnt/β-catenin, Stat3, ERK or TGFβ (68–71), which are also essential in the control of pluripotency and cellular reprogramming (72, 73). Alteration by T3 of some of these pathways, which are involved in the maintenance of a mesenchymal phenotype, is likely to be involved in stimulation of reprogramming by the hormone. In addition, future identification by RNA sequencing of a set of genes which is up-regulated or down-regulated by T3 will broaden our understanding of T3-mediated enhanced reprogramming.

AP expression is not a definitive marker of pluripotency. Furthermore, AP staining cannot distinguish partially or fully reprogrammed iPSC colonies. The induction of transcription at endogenous pluripotency loci such as *Nanog*, *Oct4* or *Sox2* during iPSC reprogramming, requires erasing of epigenetic marks characteristic of silenced genes, with histone modifications typically preceding the removal of DNA methylation marks (74). Our results show that a pure iPSC population obtained in the presence of T3 presents increased levels of expression of pluripotency genes and reduced levels of the somatic marker *Fbn2* in comparison with iPSCs obtained under control conditions, suggesting that T3 improves the properties and characteristics of the generated iPSCs. Moreover, the expression of endogenous *Sox2*, *Rex1*, *Oct4* and *Nanog* genes in T3-treated cells indeed reach levels comparable to those obtained in stablished ES cell lines. This suggests that they do not represent partially reprogrammed iPSCs but rather that they have restored a full self-renewal ES cell-like transcriptional network. This is further confirmed by the finding that the promoter regions of pluripotency genes undergo a total loss of DNA methylation showing the same pattern as ES cells. In fact, the reprogrammed MEFs were able to form EBs and to differentiate into cells of the three germ cell layers. Therefore, iPSCs generated following T3 treatment display major hallmarks of pluripotency, although *in vivo* teratoma formation and generation of viable chimeric mice would be needed to unequivocally demonstrate the fully pluripotency state of T3-induced iPSCs.

Numerous epigenetic regulators and transcription factors, including nuclear receptors, have been identified to play critical roles in reprogramming somatic cells into a pluripotent state. However, few studies for critical coactivators and corepressors are available. Transcriptional repression by the corepressor-bound receptors appears to be mediated by the recruitment of HDACs to the target genes. A repressor complex containing NCoR and HDAC3 appears to be required for repression by TR (75). In this context, it has been reported that NCoR/SMRT corepressors create an epigenetic barrier to reprogramming of MEFs obtained from Oct4-GFP transgenic mice by inducing HDAC3-dependent histone deacetylation at pluripotency loci. Corepressor knockdown appears to enhance somatic gene expression in the early phase of reprogramming, and to lead to a more potent activation of pluripotency genes at a later stage (46). In contrast with these results we found that NCoR depletion leads to a reduction of iPSC generation in the absence of hormone, which is only partially overcome by T3, indicating that the hormone is unable to compensate the lack of the corepressor and suggesting that the endogenous corepressor is required for the full promoting actions of the hormone on MEFs reprogramming. Moreover, our results indicate that the iPSC colonies formed in MEFs transduced with shNCoR are generated preferentially by cells scaping NCoR knockdown, as the iPSCs from the surviving colonies did not show an important decrease of *NCoR* transcripts. Our results also show that depletion of NCoR leads to a very significant reversion of pluripotency gene induction by T3, while having a non-statistically significant effect in the absence of hormone, again suggesting a role for NCoR on T3-mediated reprograming. In addition, we found that in pure iPSC populations there was a significant positive correlation between transcript levels of *NCoR* and of *Sox2*, *Rex1*, *Oct4* or *Nanog*. This on one hand shows that the knockdown of the corepressor rather than other possible off-target effects of the shRNA is responsible for the observed effect in iPCS generation and, on the other hand, suggests that the corepressor plays a paradoxical positive role in transcription of the pluripotency genes by T3. The mechanism responsible for downregulation of pluripotency gene expression in the absence of NCoR is presently unknown, although it is not unreasonable to think that it could involve induction of other transcriptional repressor/s and/or displacement of activating transcription factors or coactivators. In fact, NCoR binds to many nuclear receptors and to other numerous transcription factors (32), suggesting a complex role of this corepressor on transcriptional regulation. Our results also show that endogenous NCoR is required for the full induction of MEFs reprogramming by 9c-RA, as its depletion caused a marked reduction in the number of iPSC colonies generated and in the levels of pluripotency genes in the presence of this ligand. Our results do not allow to conclude that direct NCoR recruitment to thyroid hormone and retinoic acid receptors might be responsible for the stimulatory effects of their ligands on reprogramming, mainly considering that in the classical model corepressors bind the unoccupied receptors and are released in the presence of ligand (24, 32). Most likely, the reduced capacity of T3 and 9c-RA to induce reprogramming in the absence of NCoR could be related to their inability of inducing pluripotency gene expression in the absence of the corepressor. Future studies will be needed to clarify the molecular mechanism/s by which this paradoxical regulation occurs.

Class I HDACs are also recruited to the corepressor NCoR via the adaptor Sin3a protein (28). Interestingly, the Sin3a/HDAC complex is required for ES cell self-renewal (76) and contributes to maintaining pluripotency gene expression in mouse ES cells by cooperating with Nanog (77). Moreover, similar to our results with NCoR, Sin3a expression also gradually increases during the reprogramming process and Sin3a knockdown significantly reduces iPSC generation (78). Therefore, it is tempting to speculate that NCoR/Sin3a/HDAC complexes could be involved in the effects of T3 and 9c-RA on the promotion of iPSC generation described here. In addition, NCoR complexes also interact with Class IIa HDACs, which have been described to enhance mouse somatic cell reprogramming by repressing the function of the pro-mesenchymal genes and to be deleterious afterwards (79), suggesting again a complex role of corepressor complexes in acquisition and maintenance of pluripotency.

In summary, our results show that the thyroid hormone is an important component of mouse iPSC generation by assisting the Yamanaka transcription factors to reverse the changes imposed on the genome during somatic cell differentiation and to reactivate the gene network characteristic of pluripotent cells. In addition, the effect of the hormone, as well as of 9c-RA, is blocked in the absence of NCoR, indicating the requirement for the corepressor in facilitating basal reprogramming and its induction by these nuclear receptor ligands.

## Data availability statement

The original contributions presented in the study are included in the article/**Supplementary Material**. Further inquiries can be directed to the corresponding author.

## Ethics statement

The animal study was approved by Consejo Superior de Investigaciones Científicas and by the Comunidad Autónoma de Madrid Review Board (PROEX 053/15). The study was conducted in accordance with the local legislation and institutional requirements.

## Author contributions

CC-J, AM-P and RP performed the experiments. AA and AM-P wrote the manuscript. AA, SA and MF obtained funding. AA conceived and supervised the work. All authors contributed to the article and approved the submitted version.

## References

[1] Takahashi K Tanabe K Ohnuki M Narita M Ichisaka T Tomoda K. Induction of pluripotent stem cells from adult human fibroblasts by defined factors. Cell (2007) 131(5):861–72. doi: 10.1016/j.cell.2007.11.019 18035408

[2] Takahashi K Yamanaka S. Induction of pluripotent stem cells from mouse embryonic and adult fibroblast cultures by defined factors. Cell (2006) 126(4):663–76. doi: 10.1016/j.cell.2006.07.024 16904174

[3] Abad M Mosteiro L Pantoja C Canamero M Rayon T Ors I. Reprogramming in vivo produces teratomas and ips cells with totipotency features. Nature (2013) 502(7471):340–5. doi: 10.1038/nature12586 24025773

[4] Doss MX Sachinidis A. Current challenges of ipsc-based disease modeling and therapeutic implications. Cells (2019) 8(5):403. doi: 10.3390/cells8050403 31052294 PMC6562607

[5] Endoh M Niwa H. Stepwise pluripotency transitions in mouse stem cells. EMBO Rep (2022) 23(9):e55010. doi: 10.15252/embr.202255010 35903955 PMC9442314

[6] Meir YJ Li G. Somatic reprogramming-above and beyond pluripotency. Cells (2021) 10(11):2888. doi: 10.3390/cells10112888 34831113 PMC8616127

[7] Apostolou E Hochedlinger K. Chromatin dynamics during cellular reprogramming. Nature (2013) 502(7472):462–71. doi: 10.1038/nature12749 PMC4216318 24153299

[8] Bar S Benvenisty N. Epigenetic aberrations in human pluripotent stem cells. EMBO J (2019) 38(12):e101033. doi: 10.15252/embj.2018101033 31088843 PMC6576196

[9] Shakiba N Fahmy A Jayakumaran G McGibbon S David L Trcka D. Cell competition during reprogramming gives rise to dominant clones. Science (2019) 364(6438):eaan0925. doi: 10.1126/science.aan0925 30898844

[10] Wolff SC Purvis JE. Reprogramming favors the elite. Science (2019) 364(6438):330–1. doi: 10.1126/science.aax1681 31023911

[11] Li R Liang J Ni S Zhou T Qing X Li H. A mesenchymal-to-epithelial transition initiates and is required for the nuclear reprogramming of mouse fibroblasts. Cell Stem Cell (2010) 7(1):51–63. doi: 10.1016/j.stem.2010.04.014 20621050

[12] Zhuang Q Qing X Ying Y Wu H Benda C Lin J. Class iia histone deacetylases and myocyte enhancer factor 2 proteins regulate the mesenchymal-to-epithelial transition of somatic cell reprogramming. J Biol Chem (2013) 288(17):12022–31. doi: 10.1074/jbc.M113.460766 PMC3636888 23467414

[13] Cao S Yu S Li D Ye J Yang X Li C. Chromatin accessibility dynamics during chemical induction of pluripotency. Cell Stem Cell (2018) 22(4):529–42 e5. doi: 10.1016/j.stem.2018.03.005 29625068

[14] Plath K Lowry WE. Progress in understanding reprogramming to the induced pluripotent state. Nat Rev Genet (2011) 12(4):253–65. doi: 10.1038/nrg2955 PMC3273493 21415849

[15] Polo JM Anderssen E Walsh RM Schwarz BA Nefzger CM Lim SM. A molecular roadmap of reprogramming somatic cells into ips cells. Cell (2012) 151(7):1617–32. doi: 10.1016/j.cell.2012.11.039 PMC3608203 23260147

[16] Adachi K Kopp W Wu G Heising S Greber B Stehling M. Esrrb unlocks silenced enhancers for reprogramming to naive pluripotency. Cell Stem Cell (2018) 23(2):266–75 e6. doi: 10.1016/j.stem.2018.05.020 29910149

[17] Hu X Zhang L Mao SQ Li Z Chen J Zhang RR. Tet and tdg mediate DNA demethylation essential for mesenchymal-to-epithelial transition in somatic cell reprogramming. Cell Stem Cell (2014) 14(4):512–22. doi: 10.1016/j.stem.2014.01.001 24529596

[18] Ping W Hu J Hu G Song Y Xia Q Yao M. Genome-wide DNA methylation analysis reveals that mouse chemical ipscs have closer epigenetic features to mescs than oskm-integrated ipscs. Cell Death Dis (2018) 9(2):187. doi: 10.1038/s41419-017-0234-x 29416007 PMC5833453

[19] Zhao T Fu Y Zhu J Liu Y Zhang Q Yi Z. Single-cell rna-seq reveals dynamic early embryonic-like programs during chemical reprogramming. Cell Stem Cell (2018) 23(1):31–45 e7. doi: 10.1016/j.stem.2018.05.025 29937202

[20] Sridharan R Gonzales-Cope M Chronis C Bonora G McKee R Huang C. Proteomic and genomic approaches reveal critical functions of H3k9 methylation and heterochromatin protein-1gamma in reprogramming to pluripotency. Nat Cell Biol (2013) 15(7):872–82. doi: 10.1038/ncb2768 PMC3733997 23748610

[21] Papp B Plath K. Epigenetics of reprogramming to induced pluripotency. Cell (2013) 152(6):1324–43. doi: 10.1016/j.cell.2013.02.043 PMC3602907 23498940

[22] Theunissen TW Jaenisch R. Molecular control of induced pluripotency. Cell Stem Cell (2014) 14(6):720–34. doi: 10.1016/j.stem.2014.05.002 PMC4112734 24905163

[23] Bianco AC Dumitrescu A Gereben B Ribeiro MO Fonseca TL Fernandes GW. Paradigms of dynamic control of thyroid hormone signaling. Endocr Rev (2019) 40(4):1000–47. doi: 10.1210/er.2018-00275 PMC6596318 31033998

[24] Aranda A Pascual A. Nuclear hormone receptors and gene expression. Physiol Rev (2001) 81(3):1269–304. doi: 10.1152/physrev.2001.81.3.1269 11427696

[25] Aranda A Alonso-Merino E Zambrano A. Receptors of thyroid hormones. Pediatr Endocrinol Rev (2013) 11(1):2–13. 24079074

[26] Altucci L Leibowitz MD Ogilvie KM de Lera AR Gronemeyer H. Rar and rxr modulation in cancer and metabolic disease. Nat Rev Drug Discovery (2007) 6(10):793–810. doi: 10.1038/nrd2397 17906642

[27] Duong V Rochette-Egly C. The molecular physiology of nuclear retinoic acid receptors. From health to disease. Biochim Biophys Acta (2011) 1812(8):1023–31. doi: 10.1016/j.bbadis.2010.10.007 20970498

[28] Privalsky ML. The role of corepressors in transcriptional regulation by nuclear hormone receptors. Annu Rev Physiol (2004) 66:315–60. doi: 10.1146/annurev.physiol.66.032802.155556 14977406

[29] Shabtai Y Nagaraj NK Batmanov K Cho YW Guan Y Jiang C. A coregulator shift, rather than the canonical switch, underlies thyroid hormone action in the liver. Genes Dev (2021) 35(5-6):367–78. doi: 10.1101/gad.345686.120 PMC7919419 33602873

[30] Ritter MJ Amano I Imai N Soares De Oliveira L Vella KR Hollenberg AN. Nuclear receptor corepressors, ncor1 and smrt, are required for maintaining systemic metabolic homeostasis. Mol Metab (2021) 53:101315. doi: 10.1016/j.molmet.2021.101315 34390859 PMC8429965

[31] Shimizu H Astapova I Ye F Bilban M Cohen RN Hollenberg AN. Ncor1 and smrt play unique roles in thyroid hormone action in vivo. Mol Cell Biol (2015) 35(3):555–65. doi: 10.1128/MCB.01208-14 PMC4285426 25421714

[32] Perissi V Aggarwal A Glass CK Rose DW Rosenfeld MG. A corepressor/coactivator exchange complex required for transcriptional activation by nuclear receptors and other regulated transcription factors. Cell (2004) 116(4):511–26. doi: 10.1016/s0092-8674(04)00133-3 14980219

[33] Buganim Y Faddah DA Cheng AW Itskovich E Markoulaki S Ganz K. Single-cell expression analyses during cellular reprogramming reveal an early stochastic and a late hierarchic phase. Cell (2012) 150(6):1209–22. doi: 10.1016/j.cell.2012.08.023 PMC3457656 22980981

[34] Feng B Jiang J Kraus P Ng JH Heng JC Chan YS. Reprogramming of fibroblasts into induced pluripotent stem cells with orphan nuclear receptor esrrb. Nat Cell Biol (2009) 11(2):197–203. doi: 10.1038/ncb1827 19136965

[35] Zhang J Liu G Ruan Y Wang J Zhao K Wan Y. Dax1 and nanog act in parallel to stabilize mouse embryonic stem cells and induced pluripotency. Nat Commun (2014) 5:5042. doi: 10.1038/ncomms6042 25284313 PMC4205889

[36] Heng JC Feng B Han J Jiang J Kraus P Ng JH. The nuclear receptor nr5a2 can replace oct4 in the reprogramming of murine somatic cells to pluripotent cells. Cell Stem Cell (2010) 6(2):167–74. doi: 10.1016/j.stem.2009.12.009 20096661

[37] Taei A Kiani T Taghizadeh Z Moradi S Samadian A Mollamohammadi S. Temporal activation of lrh-1 and rar-gamma in human pluripotent stem cells induces a functional naive-like state. EMBO Rep (2020) 21(10):e47533. doi: 10.15252/embr.201847533 33252195 PMC7534641

[38] Wang W Yang J Liu H Lu D Chen X Zenonos Z. Rapid and efficient reprogramming of somatic cells to induced pluripotent stem cells by retinoic acid receptor gamma and liver receptor homolog 1. Proc Natl Acad Sci U.S.A. (2011) 108(45):18283–8. doi: 10.1073/pnas.1100893108 PMC3215025 21990348

[39] De Angelis MT Parrotta EI Santamaria G Cuda G. Short-term retinoic acid treatment sustains pluripotency and suppresses differentiation of human induced pluripotent stem cells. Cell Death Dis (2018) 9(1):6. doi: 10.1038/s41419-017-0028-1 29305588 PMC5849042

[40] Yang J Wang W Ooi J Campos LS Lu L Liu P. Signalling through retinoic acid receptors is required for reprogramming of both mouse embryonic fibroblast cells and epiblast stem cells to induced pluripotent stem cells. Stem Cells (2015) 33(5):1390–404. doi: 10.1002/stem.1926 PMC4863141 25546009

[41] Gonzales KA Ng HH. Driving pluripotency and reprogramming: nuclear receptors at the helm. Semin Cell Dev Biol (2013) 24(10-12):670–8. doi: 10.1016/j.semcdb.2013.07.002 23916717

[42] Liu L Luo GZ Yang W Zhao X Zheng Q Lv Z. Activation of the imprinted dlk1-dio3 region correlates with pluripotency levels of mouse stem cells. J Biol Chem (2010) 285(25):19483–90. doi: 10.1074/jbc.M110.131995 PMC2885227 20382743

[43] Chen M Zhang H Wu J Xu L Xu D Sun J. Promotion of the induction of cell pluripotency through metabolic remodeling by thyroid hormone triiodothyronine-activated pi3k/akt signal pathway. Biomaterials (2012) 33(22):5514–23. doi: 10.1016/j.biomaterials.2012.04.001 PMC3358472 22575839

[44] Percharde M Lavial F Ng JH Kumar V Tomaz RA Martin N. Ncoa3 functions as an essential esrrb coactivator to sustain embryonic stem cell self-renewal and reprogramming. Genes Dev (2012) 26(20):2286–98. doi: 10.1101/gad.195545.112 PMC3475801 23019124

[45] Ebrahimi A Sevinc K Gurhan Sevinc G Cribbs AP Philpott M Uyulur F. Bromodomain inhibition of the coactivators cbp/ep300 facilitate cellular reprogramming. Nat Chem Biol (2019) 15(5):519–28. doi: 10.1038/s41589-019-0264-z PMC6504645 30962627

[46] Zhuang Q Li W Benda C Huang Z Ahmed T Liu P. Ncor/smrt co-repressors cooperate with C-myc to create an epigenetic barrier to somatic cell reprogramming. Nat Cell Biol (2018) 20(4):400–12. doi: 10.1038/s41556-018-0047-x 29531310

[47] Sanchez A Contreras-Jurado C Rodriguez D Regadera J Alemany S Aranda A. Hematopoiesis in aged female mice devoid of thyroid hormone receptors. J Endocrinol (2020) 244(1):83–94. doi: 10.1530/JOE-19-0339 31585438

[48] Gothe S Wang Z Ng L Kindblom JM Barros AC Ohlsson C. Mice devoid of all known thyroid hormone receptors are viable but exhibit disorders of the pituitary-thyroid axis, growth, and bone maturation. Genes Dev (1999) 13(10):1329–41. doi: 10.1101/gad.13.10.1329 PMC316730 10346821

[49] Li H Collado M Villasante A Strati K Ortega S Canamero M. The ink4/arf locus is a barrier for ips cell reprogramming. Nature (2009) 460(7259):1136–9. doi: 10.1038/nature08290 PMC3578184 19668188

[50] Marti M Mulero L Pardo C Morera C Carrio M Laricchia-Robbio L. Characterization of pluripotent stem cells. Nat Protoc (2013) 8(2):223–53. doi: 10.1038/nprot.2012.154 23306458

[51] Perez RF Santamarina P Tejedor JR Urdinguio RG Alvarez-Pitti J Redon P. Longitudinal genome-wide DNA methylation analysis uncovers persistent early-life DNA methylation changes. J Transl Med (2019) 17(1):15. doi: 10.1186/s12967-018-1751 30626398 PMC6327427

[52] Redmer T Diecke S Grigoryan T Quiroga-Negreira A Birchmeier W Besser D. E-cadherin is crucial for embryonic stem cell pluripotency and can replace oct4 during somatic cell reprogramming. EMBO Rep (2011) 12(7):720–6. doi: 10.1038/embor.2011.88 PMC3128971 21617704

[53] Hunt GC Singh P Schwarzbauer JE. Endogenous production of fibronectin is required for self-renewal of cultured mouse embryonic stem cells. Exp Cell Res (2012) 318(15):1820–31. doi: 10.1016/j.yexcr.2012.06.009 PMC3582329 22710062

[54] Hanna J Saha K Pando B van Zon J Lengner CJ Creyghton MP. Direct cell reprogramming is a stochastic process amenable to acceleration. Nature (2009) 462(7273):595–601. doi: 10.1038/nature08592 19898493 PMC2789972

[55] Rais Y Zviran A Geula S Gafni O Chomsky E Viukov S. Deterministic direct reprogramming of somatic cells to pluripotency. Nature (2013) 502(7469):65–70. doi: 10.1038/nature12587 24048479

[56] Wernig M Meissner A Cassady JP Jaenisch R. C-myc is dispensable for direct reprogramming of mouse fibroblasts. Cell Stem Cell (2008) 2(1):10–2. doi: 10.1016/j.stem.2007.12.001 18371415

[57] Cvoro A Devito L Milton FA Noli L Zhang A Filippi C. A thyroid hormone receptor/klf9 axis in human hepatocytes and pluripotent stem cells. Stem Cells (2015) 33(2):416–28. doi: 10.1002/stem.1875 PMC6317531 25330987

[58] Deng C Zhang Z Xu F Xu J Ren Z Godoy-Parejo C. Thyroid hormone enhances stem cell maintenance and promotes lineage-specific differentiation in human embryonic stem cells. Stem Cell Res Ther (2022) 13(1):120. doi: 10.1186/s13287-022-02799-y 35313973 PMC8935725

[59] Hong H Takahashi K Ichisaka T Aoi T Kanagawa O Nakagawa M. Suppression of induced pluripotent stem cell generation by the P53-P21 pathway. Nature (2009) 460(7259):1132–5. doi: 10.1038/nature08235 PMC2917235 19668191

[60] Li H Collado M Villasante A Matheu A Lynch CJ Canamero M. P27(Kip1) directly represses sox2 during embryonic stem cell differentiation. Cell Stem Cell (2012) 11(6):845–52. doi: 10.1016/j.stem.2012.09.014 PMC3549496 23217425

[61] Pascual A Aranda A. Thyroid hormone receptors, cell growth and differentiation. Biochim Biophys Acta (2013) 1830(7):3908–16. doi: 10.1016/j.bbagen.2012.03.012 22484490

[62] Castillo AI Sanchez-Martinez R Moreno JL Martinez-Iglesias OA Palacios D Aranda A. A permissive retinoid X receptor/thyroid hormone receptor heterodimer allows stimulation of prolactin gene transcription by thyroid hormone and 9-cis-retinoic acid. Mol Cell Biol (2004) 24(2):502–13. doi: 10.1128/MCB.24.2.502-513.2004 PMC343792 14701725

[63] Jiang J Chan YS Loh YH Cai J Tong GQ Lim CA. A core klf circuitry regulates self-renewal of embryonic stem cells. Nat Cell Biol (2008) 10(3):353–60. doi: 10.1038/ncb1698 18264089

[64] Zhang Y Xue Y Cao C Huang J Hong Q Hai T. Thyroid hormone regulates hematopoiesis via the tr-klf9 axis. Blood (2017) 130(20):2161–70. doi: 10.1182/blood-2017-05-783043 28972010

[65] Orkin SH Hochedlinger K. Chromatin connections to pluripotency and cellular reprogramming. Cell (2011) 145(6):835–50. doi: 10.1016/j.cell.2011.05.019 PMC4858411 21663790

[66] Sridharan R Tchieu J Mason MJ Yachechko R Kuoy E Horvath S. Role of the murine reprogramming factors in the induction of pluripotency. Cell (2009) 136(2):364–77. doi: 10.1016/j.cell.2009.01.001 PMC3273494 19167336

[67] Zambrano A Garcia-Carpizo V Gallardo ME Villamuera R Gomez-Ferreria MA Pascual A. The thyroid hormone receptor beta induces DNA damage and premature senescence. J Cell Biol (2014) 204(1):129–46. doi: 10.1083/jcb.201305084 PMC3882795 24395638

[68] Alonso-Merino E Martin Orozco R Ruiz-Llorente L Martinez-Iglesias OA Velasco-Martin JP Montero-Pedrazuela A. Thyroid hormones inhibit tgf-beta signaling and attenuate fibrotic responses. Proc Natl Acad Sci U.S.A. (2016) 113(24):E3451–60. doi: 10.1073/pnas.1506113113 PMC4914168 27247403

[69] Contreras-Jurado C Alonso-Merino E Saiz-Ladera C Valino AJ Regadera J Alemany S. The thyroid hormone receptors inhibit hepatic interleukin-6 signaling during endotoxemia. Sci Rep (2016) 6:30990. doi: 10.1038/srep30990 27484112 PMC4971531

[70] Contreras-Jurado C Lorz C Garcia-Serrano L Paramio JM Aranda A. Thyroid hormone signaling controls hair follicle stem cell function. Mol Biol Cell (2015) 26(7):1263–72. doi: 10.1091/mbc.E14-07-1251 PMC4454174 25657324

[71] Lopez-Mateo I Alonso-Merino E Suarez-Cabrera C Park JW Cheng SY Alemany S. Thyroid hormone receptor beta inhibits self-renewal capacity of breast cancer stem cells. Thyroid (2020) 30(1):116–32. doi: 10.1089/thy.2019.0175 PMC6998057 31760908

[72] Ichida JK Blanchard J Lam K Son EY Chung JE Egli D. A small-molecule inhibitor of tgf-beta signaling replaces sox2 in reprogramming by inducing nanog. Cell Stem Cell (2009) 5(5):491–503. doi: 10.1016/j.stem.2009.09.012 19818703 PMC3335195

[73] Zhao H Jin Y. Signaling networks in the control of pluripotency. Curr Opin Genet Dev (2017) 46:141–8. doi: 10.1016/j.gde.2017.07.013 28806594

[74] Doege CA Inoue K Yamashita T Rhee DB Travis S Fujita R. Early-stage epigenetic modification during somatic cell reprogramming by parp1 and tet2. Nature (2012) 488(7413):652–5. doi: 10.1038/nature11333 PMC5176099 22902501

[75] Hartman HB Yu J Alenghat T Ishizuka T Lazar MA. The histone-binding code of nuclear receptor co-repressors matches the substrate specificity of histone deacetylase 3. EMBO Rep (2005) 6(5):445–51. doi: 10.1038/sj.embor.7400391 PMC1299301 15832170

[76] Zhu F Zhu Q Ye D Zhang Q Yang Y Guo X. Sin3a-tet1 interaction activates gene transcription and is required for embryonic stem cell pluripotency. Nucleic Acids Res (2018) 46(12):6026–40. doi: 10.1093/nar/gky347 PMC6158608 29733394

[77] Saunders A Huang X Fidalgo M Reimer MH Jr. Faiola F Ding J. The sin3a/hdac corepressor complex functionally cooperates with nanog to promote pluripotency. Cell Rep (2017) 18(7):1713–26. doi: 10.1016/j.celrep.2017.01.055 PMC5328122 28199843

[78] Feng J Zhu F Ye D Zhang Q Guo X Du C. Sin3a drives mesenchymal-to-epithelial transition through cooperating with tet1 in somatic cell reprogramming. Stem Cell Res Ther (2022) 13(1):29. doi: 10.1186/s13287-022-02707-4 35073971 PMC8785580

[79] Luo Z Qing X Benda C Huang Z Zhang M Huang Y. Nuclear-cytoplasmic shuttling of class iia histone deacetylases regulates somatic cell reprogramming. Cell Regener (2019) 8(1):21–9. doi: 10.1016/j.cr.2018.11.001 PMC6557759 31205685

