# Supplementary material for: The thyroid hormone enhances mouse embryonic fibroblasts reprogramming to pluripotent stem cells: role of the nuclear receptor corepressor 1
Source: Front Endocrinol (Lausanne). 2023 Dec 1;14:1235614. doi: 10.3389/fendo.2023.1235614 (PMC10722291; doi:10.3389/fendo.2023.1235614)
Supplement: Supplementary file 1 [file DataSheet_1.pdf]

Supplementary Material

The thyroid hormone enhances mouse embryonic fibroblasts reprogramming to pluripotent stem cells by a mechanism involving the Nuclear Corepressor 1

Constanza Contreras-Jurado[1,2,#], Ana Montero-Pedrazuela[1,#], Raul F. Pérez[3,4,5,6,7] Susana Alemany[1], Mario F. Fraga[3,4,5,6,7] and Ana Aranda[1,*].

*** Correspondence:** Ana Aranda, aaranda@iib.uam.es

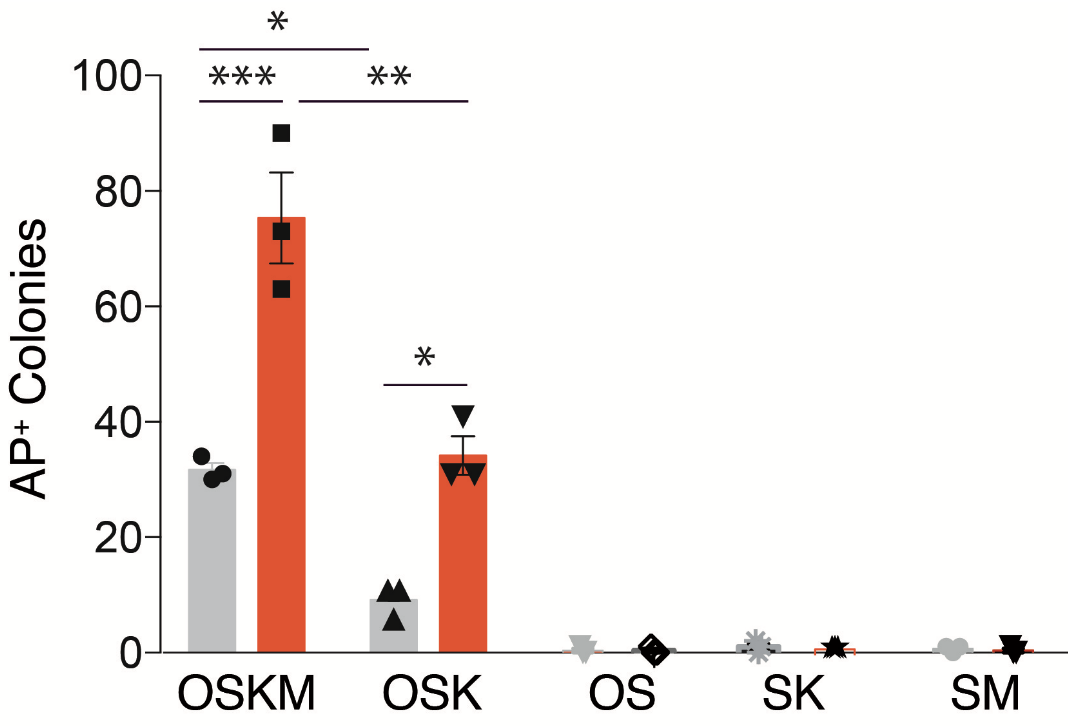

Supplementary Figure 1. The combination of two Yamanaka factors does not induce MEFs reprogramming in the presence of T3. MEFs were transduced with retroviral vectors for OSKM, OSK or with only two factors (OS, SK or SM) and incubated for 13 days in the absence and presence of T3 when the number of AP+ colonies was scored, n=3. Two-way ANOVA was used to compare OSKM and OSK conditions.

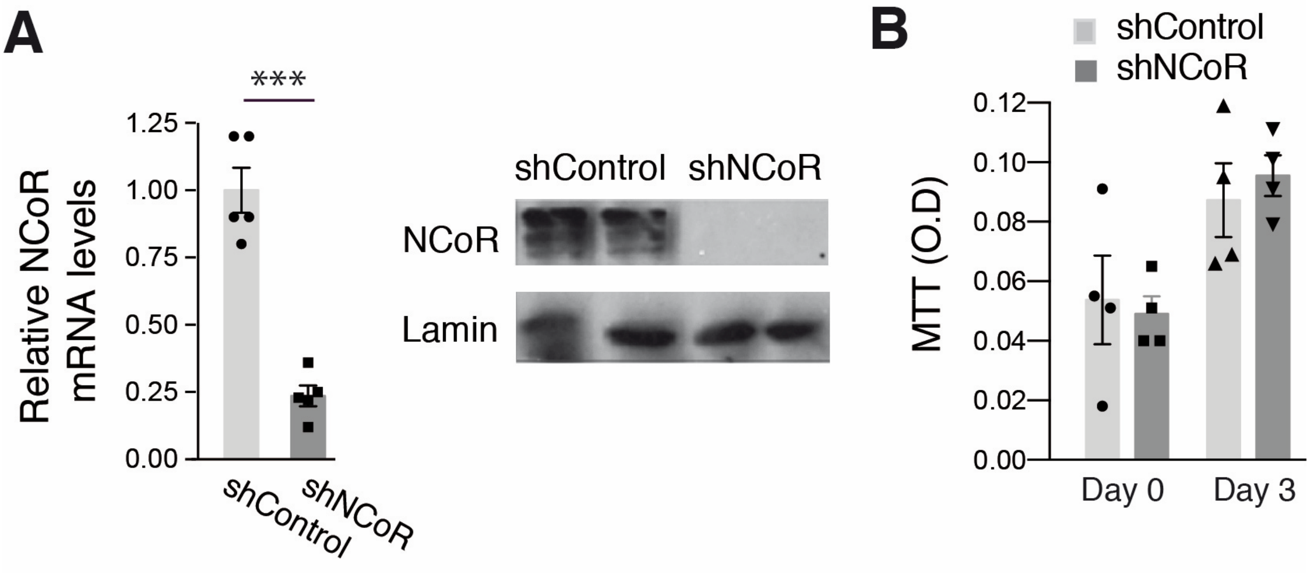

Supplementary Figure 2. NCoR silencing in OSKM-inducible MEFs. A, Efficiency of NCoR silencing in OSKM-inducible MEFs. mRNA levels (graph) and protein content (blot) of NCoR 3 days after transduction of shControl or shNCoR. Note the high efficiency of NCoR silencing. **B**, MTT analysis of cell proliferation of OSKM-inducible MEFs at days 0 and 3 after transduction with shControl or shNCoR, n=4.

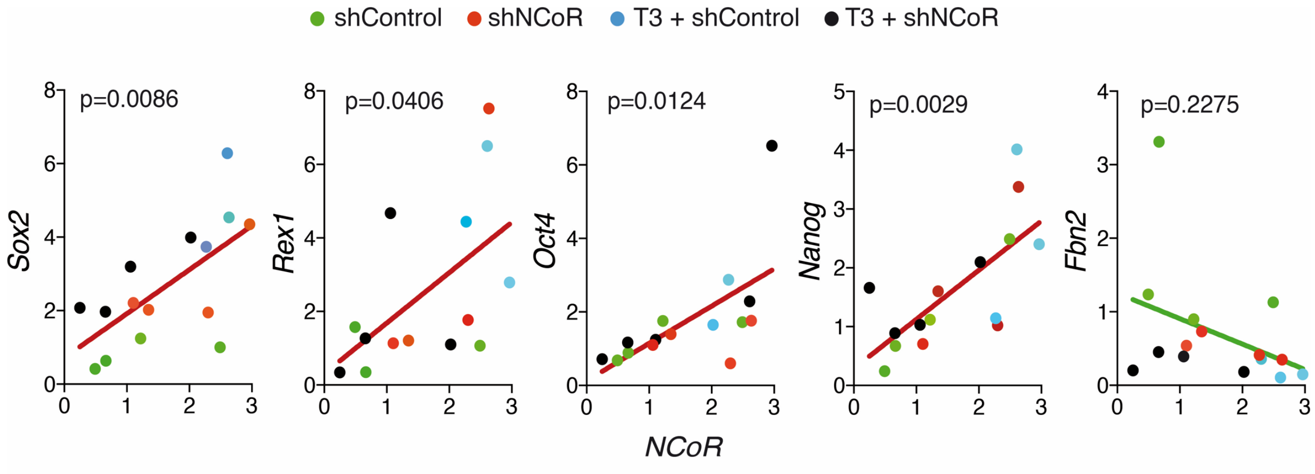

Supplementary Figure 3. Correlation between *NCoR* and pluripotency gene expression in reprogrammed MEFs. OSKM-inducible MEFs were transduced with shControl or shNCoR and incubated for 13 days with doxycycline in the presence and absence of T3. Individual colonies were then picked and expanded in the presence and absence of T3 as indicated. Transcript levels of *NCoR* obtained in the expanded colonies shown in **Fig. 6C** were plotted against those of the pluripotency genes *Sox2*, *Rex1*, *Oct4*, *Nanog* as well as *Fbn2*. The *p* values of the obtained correlations are shown.

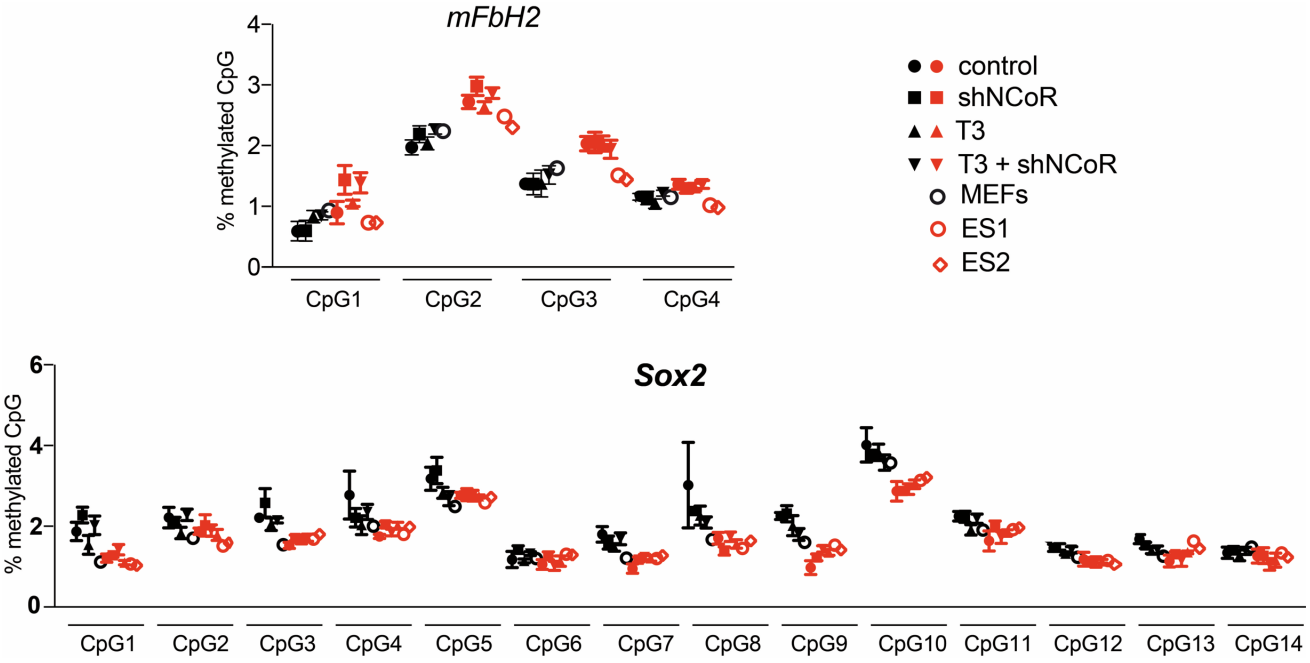

Supplementary Figure 4. Methylation analysis of the Sox2 and Fbn2 promoters. Analysis of the DNA methylation pattern of the *Sox2* and *Fbn2* promoters in non-reprogrammed MEFs (black symbols) and in the expanded iPSC colonies shown in **Fig. 6E** (red symbols) is shown. The % of CpG islands methylation in control MEFs and ES cells is also illustrated.

Supplementary Table 1. List of antibodies used

Primary antibodies

Protein	Host species	Reference and source	Dilution
Alpha-Fetoprotein	Goat	R&D Systems AF5369	1:400 IF
Alpha-SMA	Mouse	Sigma A5691	1:200 IHC
E-Cadherin (ECCD-2)	Rat	Gift of A.Cano, IIB	1:100 IF
Fibronectin	Mouse	BD Biosciences 610078	1:100 IF
Lamin B (C-20)	Goat	Santa Cruz Biotech. sc-6216	1:1,000 WB
Nanog	Rabbit	Millipore SC1000	1:300 IF
NCoR	Rabbit	Bethyl A301-145A	1:5,000 WB
Nestin	Mouse	Abcam ab6142	1:500 IF
Oct3/4	Mouse	Santa Cruz Biotech. sc-5279	1:100 IF
Tuj1 (β-tubulin III)	Mouse	Sigma T8660	1:400 IF

IHC: immunohistochemistry, IF: immunofluorescence, WB: Western Blot.

Secondary antibodies

Protein	Reference and source	Dilution
Donkey anti-goat IgG (AF 488)	Invitrogen A11055	1:500
Donkey anti-mouse IgG (AF 488)	Invitrogen A21202	1:500
Donkey anti-mouse IgG (AF546)	Invitrogen A10036	1:500
Donkey-anti-goat IgG HRP	Santa Cruz Biotech. sc-2020	1:5,000 WB
Goat-anti-mouse IgG (AF546)	Invitrogen A11030	1:500
Goat-anti-mouse IgG (AF488)	Invitrogen A11029	1:500
Goat-anti-rabbit IgG (AF488)	Invitrogen A11034	1:500
Goat-anti-rat IgG (AF546)	Invitrogen A11081	1:500
Goat-anti-rat IgG (AF647)	Invitrogen A21247	1:500
Goat-anti-rabbit IgG HRP	Santa Cruz Biotech. sc-2004	1:5,000 WB

Supplementary Table 2. Primers used for quantitative RT-PCR

	Forward primer (5'-3')	Reverse primer (5'-3')	Source
**mNCoR**	GCTGCAGGAGAGGTTTATCG	CCTGCATCTGCTGTGAGGTA	NM_011308
**Endogenous mSox2**	AAAGGAGAGAAGTTTGGAGCCCGA	GGGCGAAGTGCAATTGGGATGAAA	NM_011443
**Total mSox2**	CGTAAGATGGCCCAGGAGAA	GCTTCTCGGTCTCGGACAAA	NM_011443
**mNanog**	CAAGGGTCTGCTACTGAGATGCTCTG	TTTTGTTTGGGACTGGTAGAAGAATCAG	NM_028016
**mOct4**	GTTGGAGAAGGTGGAACCAA	CCAAGGTGATCCTCTTCTGC	NM_013633
**mRex1**	ACGAGTGGCAGTTTCTTCTTGGGA	TATGACTCACTTCCAGGGGGCACT	NM_009556
**mFbn2**	ACGCAAATCAATTCAGCAGTGT	GGTTGTCCACAGTAAGTTCCGA	NM_010181
**mGusb**	GAGGATCAACAGTGCCCATT	CAGCCTCAAAGGGGAGGT	NM_010368
**mTRα1**	GGCTGTGCTGCTAATGTCAA	CGGAGGTCAGTCACCTTCAT	NM_178060
**mTRβ**	AGACAAAGTCACCCGCAACC	CTAGCCTCTTGCTGTCATCC	NM_001113417
**p21**	AAGAGCAAAATCCGTCCCTAGC	TCATCTCAACGAAGATACAGCCA	PrimerBank ID 6755044a1
**p27**	TCAAACGTGAGAGTGTCTAACG	CCGGGCCGAAGAGATTTCTG	PrimerBank ID 31542372a1

Oct4 = Pou5f1; Rex1 = Zfp42

Supplementary Table 3. Primers and PCR conditions used for pyrosequencing

PLURIPOTENCY GENE	5´-- 3´	PCR conditions	Pyro assay
**Pyro_mOct4_F**	AGGGGTGAGAGGATTTTGAA	60 ºC, 40x	TTTGGGGTTTYGTTTTAAGGGTTGTTTTGTTTAGAYGTTTTTAATTTTYGTTTGGAAGATATAGGTAGATAGYGTTYGTTTTAG
**Pyro_mOct4_Rbio**	[Btn]CCACCCTCTAACCTTAACCT	60 ºC, 40x	
**Pyro_mOct4_S**	GGTTGAAAATGAAGGTTT	60 ºC, 40x	
**Pyro_mSox2_F**	GGGGGGGGATATAAAGGTTT	60 ºC, 45x	YGGTYGGTTGYGGGTTYGTTTTTTTYGYGYGGTTYGGGGTATAGYGTTTTGTTGGGTTYGGTTYGGYGGYGYGGTA
**Pyro_mSox2_Rbio**	[Btn]AACAACCATCCATATAATAAAAACTATCAA	60 ºC, 45x	
**Pyro_mSox2_S**	GGGGATATAAAGGTTTTTTAG	60 ºC, 45x	
**Pyro_mRex1/Zfp42_F**	TTGGGTGGGTTATTTGAAGG	60 ºC, 40x	GGTTYGTTTATATTTTYGTTTATATTTATTTTGAGYGTTTTTTATTGGGTATTTTAGTTTATTTAGTTYGGAGTTAGTTATTTYGT
**Pyro_mRex1/Zfp42_Rbio**	[Btn]CCTTAAACCCCTCCCTTTTT	60 ºC, 40x	
**Pyro_mRex1/Zfp42_S**	GGTTATTTGAAGGGTTAGG	60 ºC, 40x	
**Pyro_mFbh2/Gna11_F**	ATGTATTGTATATAGTAGGGTATGAATTTT	50 ºC, 45x	TAYGATAGGTTTGTTTAGGAATGGATGAGYGAGTYGGTTAGGGYGTTTTTTTATATTYGTAGGYGGTGAYGGTTTYGTT
**Pyro_mFbh2/Gna11_Rbio**	[Btn]CCAAAAAAAAAACTACAATTCTCTAA	50 ºC, 45x	
**Pyro_mFbh2/Gna11_S**	GGTATGAATTTTTGTTTGGTA	50 ºC, 45x	
**Pyro_mNanog_F**	TGGTGGATTTTGTAGGTGGGATTAATTG	65 ºC, 40x	GTGGGGYGTGGGTGTYGTTTGGGTGTTTGGG
**Pyro_mNanog_Rbio**	[Btn]TACCCTACCCACCCCCTATT	65 ºC, 40x	
**Pyro_mNanog_S**	ATTGTGAATTTATAGGGTTG	65 ºC, 40x	
